# Target amplicon exome-sequencing identifies promising diagnosis and prognostic markers involved in RTK-RAS and PI3K-AKT signaling as central oncopathways in primary central nervous system lymphoma

**DOI:** 10.18632/oncotarget.25463

**Published:** 2018-06-08

**Authors:** Yasuo Takashima, Yasushi Sasaki, Azusa Hayano, Jumpei Homma, Junya Fukai, Yasuo Iwadate, Koji Kajiwara, Shin Ishizawa, Hiroaki Hondoh, Takashi Tokino, Ryuya Yamanaka

**Affiliations:** ^1^ Laboratory of Molecular Target Therapy for Cancer, Graduate School for Medical Science, Kyoto Prefectural University of Medicine, Kyoto, Japan; ^2^ Center for Medical Education, Sapporo Medical University, Sapporo, Japan; ^3^ Department of Neurosurgery, Toyama Prefectural Central Hospital, Toyama, Japan; ^4^ Department of Neurological Surgery, Wakayama Medical University School of Medicine, Wakayama, Japan; ^5^ Department of Neurosurgery, Graduate School of Medical Sciences, Chiba University, Chiba, Japan; ^6^ Department of Neurosurgery, Graduate School of Medical Sciences, Yamaguchi University, Ube, Yamaguchi, Japan; ^7^ Department of Pathology, Toyama Prefectural Central Hospital, Toyama, Japan; ^8^ Research Institute for Frontier Medicine, Sapporo Medical University, Sapporo, Japan

**Keywords:** primary central nervous system lymphoma, somatic mutation, copy number variation, RAS signaling, prognosis

## Abstract

Exome-sequencing for somatic mutation detection and copy number variation analysis are effective and valid methods for evaluating human cancers in current molecular medicine. We conducted target amplicon exome-sequencing analyses using PCR target enrichment and next-generation sequencing on Ion Proton semiconductor sequencers. Twenty-seven primary central nervous system lymphoma (PCNSL) specimens and their corresponding noncancerous tissues were used for multiplex PCR amplification to obtain targeted coverages of the entire coding regions of 409 cancer-related genes. The average of the total numbers of somatic mutations including single-nucleotide variations and insertion/deletion mutations in each specimen was 13.3. Of these, the average of the ratios of nonsynonymous substitutions in each specimen was 74.8%. The most frequent mutations in 27 specimens were in *PIM1, MYD88, CD79B, DST, IRF4, ERBB3, MYH11, DCC*, and *KMT2D.* Furthermore, somatic mutations of *MYH11* were related to poor prognoses in PCNSL patients. Copy number variations were also duplicated and/or deleted from deep-sequencing in segmental genomic islands. In addition to these prognostic marker candidates, analysis of RTK-RAS-MAPK signaling and the PTEN-PI3K-AKT proapoptotic pathway showed that somatic activations and aberrations, respectively, may be involved in a promising central oncopathway harboring mTOR, c-Myc, FOXO1, and p53. This study provides a foundation for molecular targeted therapies based on genome diagnostics and prognosis in PCNSL.

## INTRODUCTION

Primary central nervous system lymphoma (PCNSL) is a rare subgroup of diffuse large B-cell lymphoma (DLBCL) arising in the brain, meninges, spinal cord, and eyes [1, 2] and is an aggressive malignant variant of nodal non-Hodgkin lymphoma (NHL) localized in the central nervous system. This type of lymphoma accounts for 3% of all primary brain tumors and 1% of NHL in adults [1, 3]. Most PCNSLs (90%) are also immune-privileged site-associated DLBCLs according to the World Health Organization diagnostic criteria [1]. Despite intensive treatments including high-dose methotrexate based on poly-chemotherapy with whole brain radiotherapy, the median overall survival (OS) is approximately 4 years and shows a poorer prognosis than extracerebral DLBCL [3–5].

High-resolution genomic arrays and whole genome sequencing have been shown to be efficient approaches for inclusive analyses of chromosome imbalances and gene mutations in solid cancers and hematologic malignancies, including DLBCL [6–8]. The molecular biology and medicine of PCNSL have been partially determined by array-based profiling of genomic and transcriptional alterations [9–15]. Recent studies have reported that droplet digital PCR in liquid biopsies detected the MYD88 mutation L265P in cerebrospinal fluid [16]. The latest next generation sequencing (NGS) also has detected the non-invasive somatic mutations in PCNSL [17] and vitreoretinal lymphomas from small-volume intraocular liquid biopsies with *de novo* methods for targeted therapies [18]. In PCNSL, *BCL6* overexpression and aberrant somatic hypermutation of many genes, coupled with surface localization of IgM, suggest that the tumors are arrested at the terminal B-cell differentiation stage [19]. Studies of chromosomal copy number variation (CNV) have identified recurrent CNVs in PCNSL, and exome-sequencing analysis revealed that the *CD79B, MYD88, TBL1XR1*, and *ODZ4* genes are most frequently mutated [14, 20, 21], in addition to *PRDM1* and *CARD11* [22, 23].

Additionally, recent studies have indicated that molecular changes stimulate NF-κB signaling and sustain the high-breakpoint cluster region [24, 25], as well as downregulate genes within minimal regions of imbalances including HLA class II genes at deleted 6p21, losses of 6q, 8q12, and 9p21, and gains of 7q, 11q, and chromosome 12 [9, 26, 27]. In contrast, pathogenetic insights are mainly derived by locus-specific approaches using fluorescence *in situ* hybridization and sequencing of candidate genes identified as recurrently translocated genes such as *BCL6* [28–30] and recurrently mutated genes such as *MYD88* and *CD79B* [31, 32].

These results demonstrated that gene mutations including an aberrant somatic hypermutation and specified CNVs influence the malignancies of PCNSL cells. However, few retrospective studies have examined the detailed molecular network and cell signaling based on diagnosis with gene mutations and CNVs or the prognosis of patients with PCNSL. Thus, in this study, we conducted target amplicon exome-sequencing analyses using PCR target enrichment and next-generation sequencing to obtain targeted coverage of the entire coding regions of 409 cancer-related genes in the genomic landscape by using tumor specimens and matched normal control tissues derived from 27 patients with PCNSL. We identified significant single-nucleotide variations (SNVs) in myosin heavy chain 11 (*MYH11*) associated with poor prognosis. Furthermore, CNVs of the 12 prognostic marker candidates and genes related to RTK-RAS-MAPK signaling and the PTEN-PI3K-AKT proapoptotic pathway were examined, and their biological significance was confirmed by survival analysis of patients with PCNSL.

## RESULTS

### Targeted amplicon sequencing of PCNSL

We performed semi-conductor sequencing of all exons of 409 cancer-related genes in 27 PCNSL specimens and 17 matched normal control tissues using the Ion Ampliseq Comprehensive Cancer Panel (Supplementary Figures 1 and 2). In this study, somatic mutations were detected in 136 of 409 genes (33.25%, variant frequency >15%) (Supplementary Figure 2). The sequencing overview including reads, coverage, and uniformity of the read coverage distribution is shown in Supplementary Figure 3. Each specimen underwent an average 4.88 million sequencing reads after quality filtering. The averages of percent (%) reads of targets in tumors, matched paired normal tissues, and total specimens were 97.5 (range: 94.6–98.7), 98.1 (92.4–99.1), and 97.7 (92.4–99.1). Coverage depths were 286.5 (145.0–467.9), 345.8 (100.9–507.6), and 309.4 (100.9–507.6), respectively. The percent 20x coverage in total specimens was 96.5 (91.7–99.0).

### Identification of frequent somatic mutations in 136 cancer-related genes in PCNSL

**Figure 1:**
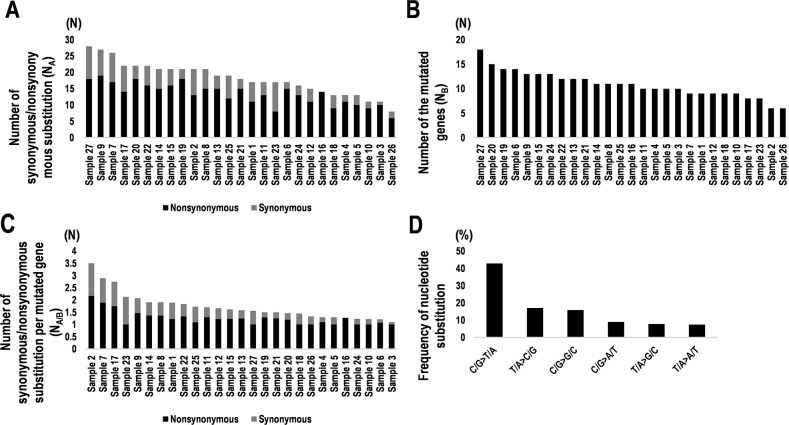
Summary of synonymous and nonsynonymous mutations and nucleotide substitutions in 27 PCNSL specimens **(A)** Numbers of synonymous and nonsynonymous mutations in PCNSL specimens (N_A_). Black and gray bars indicate nonsynonymous and synonymous substitutions, respectively. **(B)** Numbers of genes containing mutations in PCNSL specimens (N_B_). **(C)** Numbers of synonymous and nonsynonymous mutations per gene (N_A/B_). Number of synonymous/nonsynonymous substitutions (N_A_) were divided by the number of mutated genes (N_B_). Nonsynonymous (black) and synonymous substitutions (gray). **(D)** Frequencies of types of nucleotide substitutions in PCNSL specimens.

Somatic mutations including SNVs and insertion and deletion mutations (INDELs) were identified by tumor-normal analyses in which germline variants can be eliminated from the tumor variants. The sequencing results derived from 27 PCNSL specimens were compared with the results from 17 paired normal control tissues and control sequence data within Ion Reporter software (see Methods). All SNVs and INDELs identified were confirmed by visual inspection with the Integrative Genomics Viewer (IGV). In total, 361 somatic mutations in 136 genes were identified. Averages of nonsynonymous and synonymous mutations were 13.37 (range: 6–19) and 4.77 (0–10), respectively, per specimen (Figure 1A). The ratio of nonsynonymous mutation was 74.8% (47.0–100) (Figure 1A). The average number of mutated genes was 10.85 (6–18) per specimen (Figure 1B); thus, the frequency of nonsynonymous mutations in the mutated genes per specimen was 1.25 (1.0–2.16), whereas that of synonymous mutations was 0.47 (0–1.33), which is statistically nearly no synonymous mutation per specimen (Figure 1C). The most common nucleotide substitutions were C/G>T/A transitions accounting for 42.85% of substitutions, whereas T/A>C/G transitions were 17.08% (Figure 1D). In contrast, C/G>A/T and T/A>G/C transversions accounted for only 9.0% and 7.76% of substitutions (Figure 1D).

### Genes recurrently affected by protein-coding aberration in PCNSL

**Figure 2:**
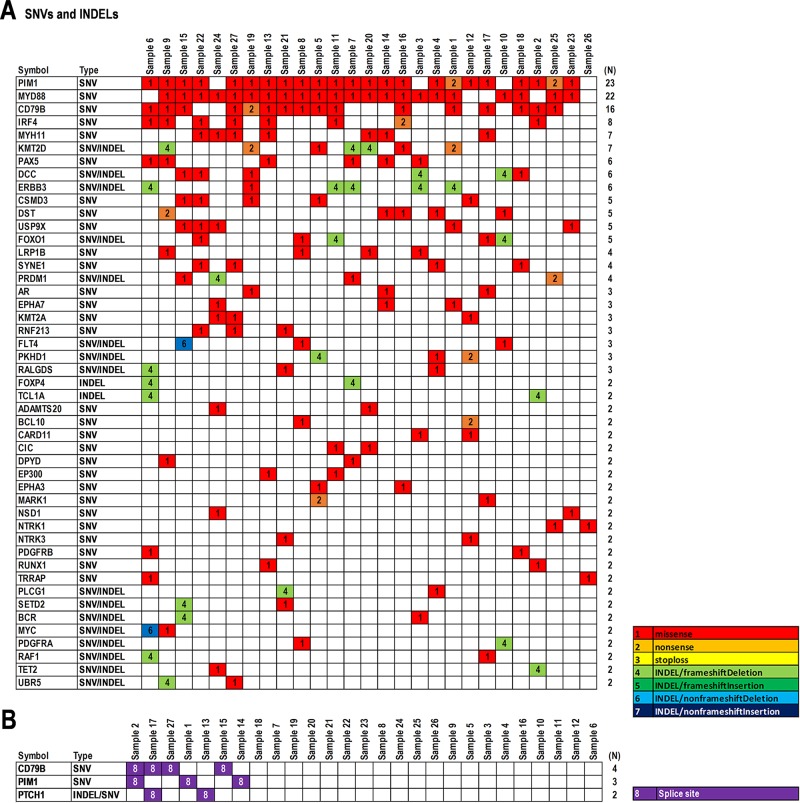
Summary of somatic mutations in 27 PCNSL specimens **(A)** Representative SNVs and INDELs detected in PCNSL specimens (N ≥ 2). **(B)** Splice site mutations detected in PCNSL specimens (N ≥ 2). Mutation types are shown in the data matrix as missense, nonsense, stop-loss, INDELs including frameshift/nonframeshift with/without deletion or insertion, and splice site mutations, as per the color configuration panel. Numbers on the right side of panels (N) indicate the numbers of specimens.

Twenty-five genes harboring somatic mutations including SNVs and INDELs with total count more than two per gene in the 27 PCNSL specimens were found (Supplementary Figure 4A and 4B). Particularly, proto-oncogene serine/threonine (Ser/Thr)-protein kinase 1 (*PIM1*) (N = 71), myeloid differentiation primary response 88 (*MYD88*) (N=22), cluster of differentiation 79B (*CD79B*) (N = 21), and interferon regulatory factor 4 (*IRF4*) (N = 13) were most frequent, followed by *ERBB3* (N = 8), *KMT2D* (N = 8), *MYH11* (N = 7), *DST* (N = 7), *CSMD3* (N = 6), *DCC* (N = 6), *FOXO1* (N = 5), *PAX5* (N = 5), and *USP9X* (N = 5) (Supplementary Figure 4A and 4B). A total of 136 genes were affected by mutations in exons (Figure 2A, and Supplementary Figures 5 and 6A). The genes harboring the most frequent somatic mutations were *PIM1* (85.1%), *MYD88* (81.4%), *CD79B* (59.2%), *IRF4* (29.6%), *MYH11* (25.9%), *KMT2D* (25.9%), *PAX5* (22.2%), *DCC* (22.2%), and *ERBB3* (22.2%) (Figure 2A). Splice site mutations were also detected in *CD79B* (14.8%), *PIM1* (11.1%), and patched-1 (*PTCH1*) (7.4%) (Figure 2B and Supplementary Figure 6B). These results indicate that *PIM1, MYD88*, and *CD79B* had the most frequent somatic mutations in PCNSL, and *IRF4, MYH11, PAX5*, and *DCC* may be also considered as candidate diagnostic markers.

**Figure 3:**
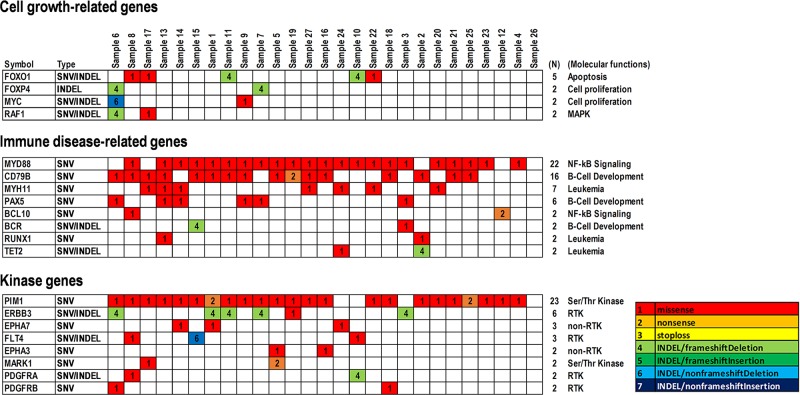
Summary of significant SNVs and INDELs detected in genes related to cell growth and immune disease and kinase genes in PCNSLs Representative SNVs and INDELs are shown (N ≥ 2). Cell growth-related genes, including cell proliferation, MAP-kinase, and apoptosis (upper). Immune disease-related genes, including NF-κB signaling, leukemia, and B-cell development and differentiation (middle). Kinase genes, including receptor tyrosine kinase (RTK), non-RTK, and Ser/Thr kinase (lower). Mutation types are shown in the data matrix as missense, nonsense, stop-loss, and INDEL mutations including frameshift/non-frameshift with/without deletion or insertion, as per the color configuration panel. Numbers (N) at the right side of the panels indicate the numbers of specimens detected.

In contrast, reconstructing the data matrix for somatic mutations including SNVs and INDELs showed a bias for molecular functions and cellular signaling pathways (Figure 3 and Supplementary Figure 7). Somatic mutations were found in cell growth-related genes, such as *FOXO1* (18.5%) in apoptosis, *FOXP4* (7.4%) and *MYC* (7.4%) in cell proliferation, and *RAF1* (3.7%) in MAP-kinase (Figure 3). Further, somatic mutations in genes related to immune disease signaling pathways and kinase genes were found (Figure 3). As immune disease-related genes, *MYD88* (81.4%) and *BCL10* (25.9%) in NF-ĸB signaling, *CD79B* (59.2%), *PAX5* (22.2%), and *BCR* (7.4%) in B-cell development, and *MYH11* (25.9%), *RUNX1* (7.4%), and *TET2* (7.4%) in leukemia were observed (Figure 3). Similarly, *PIM1* (85.1%) and *MARK1* (7.4%) in Ser/Thr-kinases, *ERBB3* (22.2%), *FLT4* (11.1%), *PDGFRA* (7.4%), and *PDGFRB* (7.4%) in receptor tyrosine kinases (RTKs), and *EPHA7* (11.1%) and *EPHA3* (7.4%) in non-RTKs were found in kinase genes (Figure 3). These results suggest that the above-mentioned genes harboring somatic hypermutations including *MYD88*, *CD79B*, *MYH11*, *PIM1*, and *ERBB3* are not directly involved in the cell cycle, but rather in immune disease signaling pathways and phosphorylation of proteins such as RTKs, non-RTKs, and Ser/Thr-kinases. In addition, *BCL10, BCR, RUNX1, EPHA3/7, FLT4, MARK1*, and *PDGFRA/B* may also be considered as candidates for genome diagnostics in PCNSL. Of these, representative mutations within *BCL10, CD79B, MYD88, MYH11, PAX5*, and *TET2*, as immune disease-related genes, were also validated with Sanger sequencing (Supplementary Figure 8A and 8B).

### Correlation between patient's survivals and candidate markers for genome diagnosis

We examined the correlation between PCNSL patient survivals and candidates of markers for genome diagnostics by calculating the hazard ratio (HR) and using the Kaplan-Meier method (Supplementary Figure 9A and 9B). The median age of the 27 PCNSL patients was 66 years (range, 31–85 years). A total of 13 patients were male (48.1%) and 14 patients were female (51.8%). The median survival time was 59.46 months (95% confidence interval (CI): 25.13–73) and the overall survival (OS) status was “deceased” in 18 (66.6%) and “living” in 9 patients (33.3%) at the last follow-up. Univariate analyses for OS were performed according to age and gender. The HR for age ≥50 years tended to be higher than that for age <50 years (HR = 1.76, 95%CI: 0.5–11.17, P = 0.42), and the HR for females tended to be slightly lower than that for males (HR = 0.92, 95%CI: 0.35–2.37, P = 0.86). However, no significant differences were observed in each subgroup analysis because of the small number of samples and the biases.

**Figure 4:**
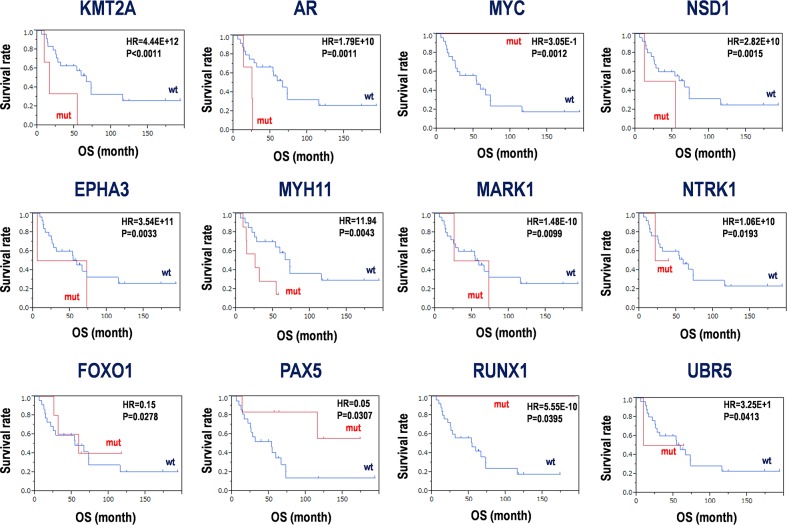
Candidates for prognosis markers derived from SNV and INDELs in PCNSLs Kaplan-Meier analysis for the 12 candidate genes was performed. Hazard ratios (HR) and P-values with log-rank test are shown in each panel. mut; mutation, wt; wild-type, OS; overall survival.

The HRs for the above-mentioned 136 genes were calculated between the subgroup harboring mutations and wild-types (Table 1 and Figure 4). As a result, 12 genes including *KMT2A* (HR = 4.44×10^12^, P < 0.0011), *AR* (HR = 1.79×10^10^, P = 0.001), *MYC* (HR = 0.305, P = 0.0012), *NSD1* (HR = 2.82×10^10^, P = 0.0015), *EPHA3* (HR = 3.54×10^11^, P = 0.0033), *MYH11* (HR = 11.94, P = 0.0043), *MARK1* (HR = 1.48×10^-10^, P = 0.0099), *NTRK1* (HR = 1.06×10^10^, P = 0.0193), *FOXO1* (HR = 0.15, P = 0.0278), *PAX5* (HR = 0.05, P = 0.0307), *RUNX1* (HR = 5.55×10^-10^, P = 0.0395), and *UBR5* (HR = 32.5, P = 0.0413) were detected (Table 1). Kaplan-Meier curves were also clearly divided (Figure 4). Somatic mutations in *KMT2A, AR, NSD1, EPHA3, MYH11, NTRK1*, and *UBR5* showed poor prognoses, while those in *MYC, MARK1, FOXO1, PAX5*, and *RUNX1* showed good prognoses, with significant differences between mutant and wild-type groups. However, it is considered that a small sample size and biases of sample numbers between two subgroups with or without somatic mutations resulted in discrepancies between hazard ratios and their probabilities. Thus, thresholds of P≈0.05 might be false positives. Therefore, in the study, only *KMT2A*, *AR*, *MYC*, *NSD1*, *EPHA3*, *MYH11*, and *MARK1* with P < 0.01 should be distinct as candidates for diagnosis and prognosis markers from the other candidates with relatively high P values.

**Table 1:** Candidates for prognostic markers based on somatic mutations in PCNSL

Symbol	Refseq	Description	Alias	HR	P-value
KMT2A	NM_005933	Homo sapiens lysine methyltransferase 2A (KMT2A), transcript variant 2	KMT2A, ALL-1, CXXC7, HRX, HTRX1, MLL, MLL/GAS7, MLL1, MLL1A, TET1-MLL, TRX1, WDSTS, MLL-AF9, lysine methyltransferase 2A, Histone-lysine N-methyltransferase HRX	4.44E+12	<0.0011
AR	NM_001011645	Homo sapiens androgen receptor (AR), transcript variant 2	AR, AIS, AR8, DHTR, HUMARA, HYSP1, KD, NR3C4 (nuclear receptor subfamily 3, group C, member 4), SBMA, SMAX1, TFM, androgen receptor	1.79E+10	0.0011
MYC	NM_002467	Homo sapiens MYC proto-oncogene, bHLH transcription factor (MYC), transcript variant 1	MYC, MRTL, MYCC, bHLHe39, c-Myc, v-myc avian myelocytomatosis viral oncogene homolog, MYC proto-oncogene, bHLH transcription factor	3.05E-01	0.0012
NSD1	NM_172349	Homo sapiens nuclear receptor binding SET domain protein 1 (NSD1), transcript variant 1	STO	2.82E+10	0.0015
EPHA3	NM_005233	Homo sapiens EPH receptor A3 (EPHA3), transcript variant 1	EPHA3, Epha3, AW492086, Cek4, ETK1, End3, Hek, Hek4, Mek4, Tyro4, EK4, ETK, EPH receptor A3, HEK, HEK4, TYRO4	3.54E+11	0.0033
MYH11	NM_001040114	Homo sapiens myosin heavy chain 11 (MYH11), transcript variant SM1B	MYH11, AAT4, FAA4, SMHC, SMMHC, myosin, heavy chain 11, smooth muscle, myosin heavy chain 11	11.94	0.0043
MARK1	NM_001286124	Homo sapiens microtubule affinity regulating kinase 1 (MARK1), transcript variant 1	MARK1, MARK, Par-1c, Par1c, microtubule affinity regulating kinase 1	1.48E-10	0.0099
NTRK1	NM_002529	Homo sapiens neurotrophic receptor tyrosine kinase 1 (NTRK1), transcript variant 2	NTRK1, MTC, TRK, TRK1, TRKA, Trk-A, p140-TrkA, neurotrophic receptor tyrosine kinase 1	1.06E+10	0.0193
FOXO1	NM_002015	Homo sapiens forkhead box O1 (FOXO1)	FOXO1, FKH1, FKHR, FOXO1A, forkhead box O1	0.15	0.0278
PAX5	NM_001280547	Homo sapiens paired box 5 (PAX5), transcript variant 2	PAX5, ALL3, BSAP, paired box 5	0.05	0.0307
RUNX1	NM_001001890	Homo sapiens runt related transcription factor 1 (RUNX1), transcript variant 2	RUNX1, AML1, AML1-EVI-1, AMLCR1, CBF2alpha, CBFA2, EVI-1, PEBP2aB, PEBP2alpha, runt related transcription factor 1	5.55E-10	0.0395
UBR5	NM_001282873	Homo sapiens ubiquitin protein ligase E3 component n-recognin 5 (UBR5), transcript variant 2	UBR5, DD5, EDD, EDD1, HYD, ubiquitin protein ligase E3 component n-recognin 5	3.25E+01	0.0413

Furthermore, SNVs and INDELs were analyzed by SIFT and PolyPhen-2 (Supplementary Table 1) and mapped onto functional domains into the genes (Supplementary Figure 10A and 10B). The average SIFT score is 0.006 (range: 0-0.05) and the average PolyPhen-2 score is 0.895 (0.151-1) in 127 somatic mutations including SNVs and INDELs with allele frequency >15%. As for representative mutations, the results from SIFT and PolyPhen-2 analyses were summarized in Supplementary Table 1. Although a part of mutations was evaluated, the most of data showed <0.05 in SIFT score and >0.15 in PolyPhen-2 score, suggestive of significant mutations in protein structures and functions.

Of these, some SNVs were identical in multiple PCNSL specimens. For example, 896delC (7.4%) in *FOXO1*, 7825C>G (7.4%) in *KMT2A*, 845C>G (18.5%) in *MYH11*, 4165T>C (7.4%) in *NSD1*, and 2303C>T (7.4%) in *NTRK1* were detected (Supplementary Figure 10A). Significant mutations are also located in functional domains, such as the ligand-binding domain of hormone receptors, HOLI domain of AR (Val717Ile; 3.7% and Arg775His; 3.7%), sterile-α motif (SAM) domain (Gln962Lys; 3.7%) and tyrosine kinase catalytic (TyKc) domain (Ser768Leu; 3.7%) of EPHA3, plant homeodomain (PHD) finger of KMT2A (Pro115His; 3.7%), myosin large ATPases of MYH11 (Ala282Gly; 18.5%, Pro82His; 3.7%, and Asn282Lys; 3.7%), TyKc domain of NTRK1 (Pro768Leu; 7.4%), and Runt domain for DNA-binding and protein-protein interaction (Ile114Met; 3.7% and Leu472Pro; 3.7%) of RUNX1 (P < 5.01×10^-8^) (Supplementary Figure 10B). Interestingly, a few single nucleotide polymorphisms (SNPs), such as rs137852572 and rs201747706, were also included in *AR* and *RUNX1*, respectively (Supplementary Figure 10B). These results indicate that the 12 candidates are associated with SNVs/INDELs and prognoses, and 6 candidates, *AR, EPHA3, KMT2A, MYH11, NTRK1*, and *RUNX1*, harbor significant mutations in the functional domains of the proteins, which may be useful for genome diagnostics and prognostic predictions in PCNSL. Combined with the both data from Kaplan-Meier analysis and domain mapping of somatic mutations, the mutations in the four genes including *AR, EPHA3, KMT2A*, and *MYH11* with significant hazard ratios for prognosis and domain mutations could be considered as candidates for genomic diagnosis and prognosis markers in PCNSL in the study.

### CNVs of prognostic marker candidates

**Figure 5:**
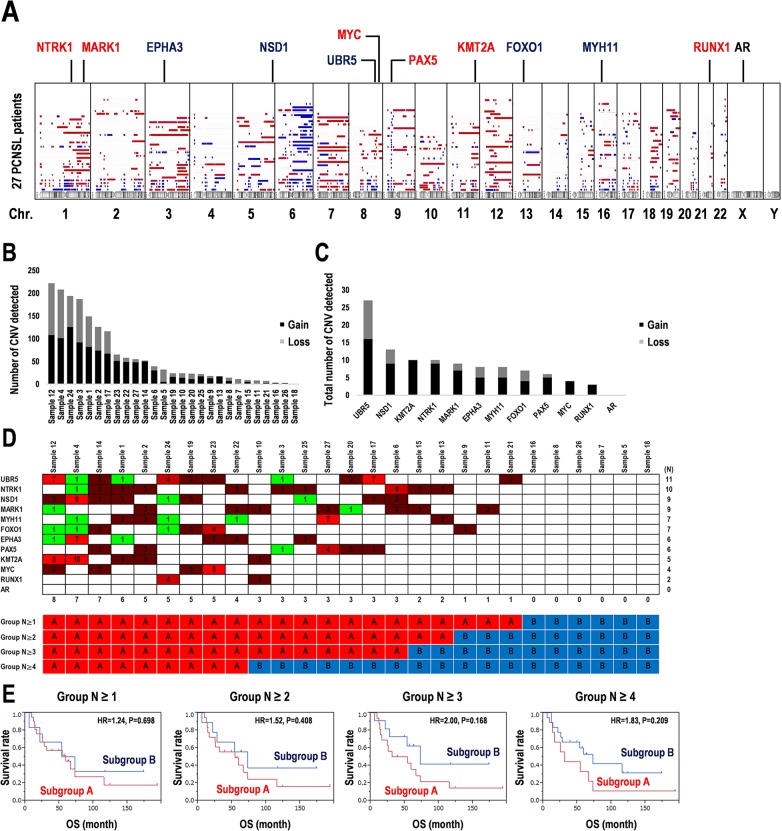
Copy number variations detected in 12 prognostic marker candidates in 27 PCNSL specimens **(A)** Copy number variations (CNVs) over the entire genome were visualized in karyotype view. Genomic positions of 12 candidates of prognosis markers were mapped. The regions of CNVs with gain (red) and loss (blue) indicate as colored bars and gene names. **(B)** Total numbers of the regions detected with significant CNVs in each sample. **(C)** Total numbers of the regions detected with significant CNV regions including the 12 candidate of prognosis markers. **(D)** CNVs detected into the region including the 12 candidate prognostic markers in each PCNSL specimen. Numbers in the matrix indicate CNV > 2: Gain (red) and < 2: Loss (green) (upper). Numbers at the bottom of the upper panel indicate the numbers of genes associated with CNVs in each PCNSL specimen. The 27 PCNSL specimens were divided into the two subgroups according to the CNV frequency per specimen after two-way clustering (lower). **(E)** Kaplan-Meier survival analysis for the combination of the 12 candidate genes associated with SNVs and CNVs. Analyses were performed according to the groups as shown in **D**. OS; overall survival time, HR; hazard ratio.

We next examined copy number variations (CNVs) in the 27 PCNSL specimens were mapped to human chromosomes by copy number gains and losses (Figure 5A, see Methods). The averages of the numbers of regions detected by gains and losses of the copy number per specimen were 37.85 (range: 1–126) and 25.74 (0–114), respectively (Figure 5B). Additionally, averages of copy numbers per 100 Mb per chromosome in gains, losses, and total were 36.7 (0–116.4), 25.1 (0–97.5), and 61.5 (0–69.4), respectively (Supplementary Figure 11A). Particularly, no CNVs were detected in the sex chromosomes X and Y (Supplementary Figure 11A). In contrast, the ratios of gains in copy number per 100 Mb were high in chromosomes 21 (100%), 12 (83.6%), 19 (78.3%), and 7 (75.9%), while the ratio of the loss of the copy number per 100 Mb was high in chromosome 6 (70.9%) (Supplementary Figure 11B).

The 12 prognostic marker candidates shown in Table 1 and Figure 4 were also mapped as shown in Figure 5A. Whether the CNVs were increased or decreased was also examined (Figure 5C). Most copy number alterations in *NTRK1, MARK1, MYC, PAX5, KMT2A*, and *RUNX1* were increased (gain > 70%). In contrast, copy number losses in *EPHA3, NSD1, UBR5, FOXO1*, and *MYH11* were also found (loss > 30%) (Figure 5C, Supplementary Figure 12). The CNV was not detected in *AR* (Figure 5C, Supplementary Figure 12). Second, among the 12 candidates, 27 PCNSL specimens were divided into subgroups associated with or without CNVs according to CNV frequencies (Figure 5D, Supplementary Figure 13A). Kaplan-Meier analysis demonstrated that the subgroup associated with the CNVs has a poor prognosis compared to that without the CNVs (Group N ≥ 1, HR = 1.24, log-rank test: P = 0.698) and that with 2-4 of CNVs (Group N ≥ 2: HR = 1.52, P = 0.408, Group N ≥ 3: HR = 2.00, P = 0.168, Group N ≥ 4, HR = 1.838, P = 0.209) (Figure 5E, Supplementary Figure 13B). Especially, differential CNV combinations of *EPHA3, KMT2A, PAX5*, or *RUNX1* between both subgroups tended to divide the survival curves of the two subgroups (Group N ≥ 3: HR = 2.00, P = 0.168) (Figure 5D and 5E, Supplementary Figure 13A and 13B). Using many CNVs (N ≥ 5) did not divide into the balanced two groups in the sample numbers. Thus, the analysis for the CNV combinations were limited by the subgroups with a small number of CNVs. Consequently, the significances of the 12 candidates as prognostic markers were validated not only by their gene mutations but also by CNV combinations, which may also be useful for genome diagnostics in PCNSL. However, the optimized combination of the 12 candidates for genome diagnostics requires further analyses in a larger population.

### Altered signaling pathways associated with CNVs in PCNSL

**Figure 6:**
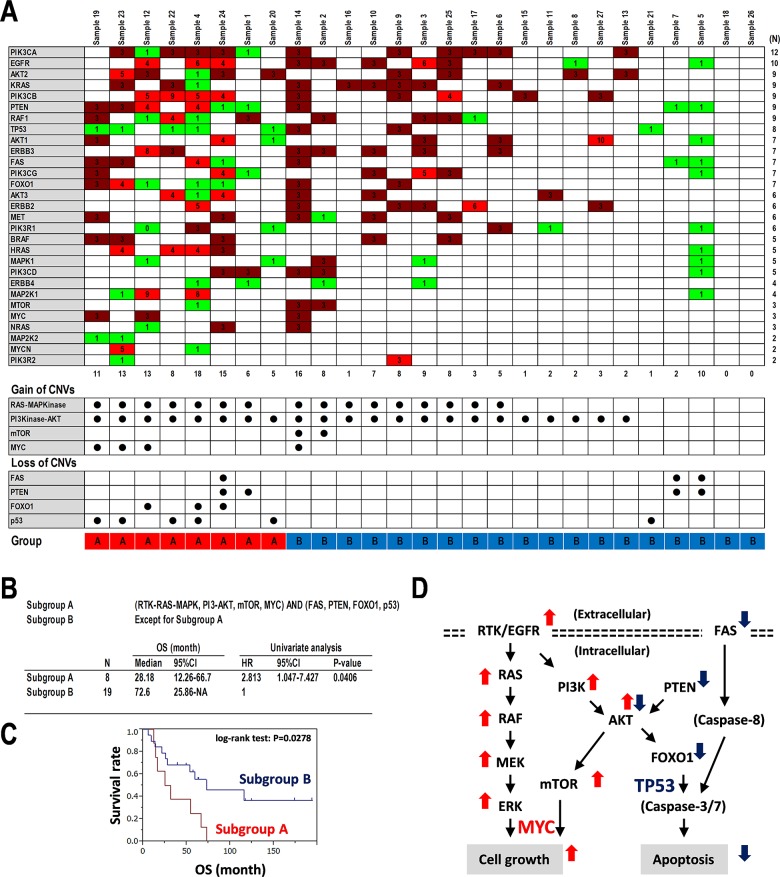
Copy number variations detected in oncopathways **(A)** Copy number variations (CNVs) detected in regions including genes related to RTK-RAS-MAPK, mTOR, MYC, PTEN-PI3K-AKT, FAS, FOXO1, and p53 pathways in each PCNSL specimen. Numbers in the matrix indicate CNV (top panel). CNV > 2: Gain (red), CNV < 2: Loss (green) (upper). Numbers at the bottom of the upper panel indicate the numbers of genes associated with CNVs in each PCNSL specimen. CNVs for upregulated including RAS-MAPK, PI3K-AKT, mTOR, and MYC and downregulated pathways including p53, FOXO1, PTEN, and FAS are summarized (middle and bottom panels). Closed circles indicate “detected.” **(B-C)** Statistics and Kaplan-Meier survival analysis for upregulated pathways including RAS-MAPK, PI3K-AKT, mTOR, and MYC and downregulated pathways including p53, PTEN, FOXO1, and FAS. 27 PCNSL specimens were divided into two subgroups with or without the CNVs in the gains of defined oncopathways and losses of defined proapoptotic pathways after two-way clustering, as shown at the bottom of the data matrix in A. Group A: Harboring CNVs for upregulated pathways including RAS-MAPK, PI3K-AKT, mTOR, or MYC and downregulated pathways including p53, PTEN, FOXO1, or FAS, Group B: Except for Group A. (B) Definition of groups, and statistics for OS and univariate analysis for HR. (C) Kaplan-Meier analysis. **(D)** Schematics of RTK-RAS-MAP-kinase and PI3-kinase-AKT pathways. RTK; receptor tyrosine kinase, double dotted lines; plasma membranes. Thin arrows; signal direction, thick arrows, upregulated (red) and downregulated (blue).

Here, we focused on CNVs of kinase genes including the RTK-RAS-MAP-kinase and PI3-kinase-AKT pathways, as few somatic mutations were detected in cell growth-related genes, while kinase genes including RTK, non-RTK, Ser/Thr-kinase, and PI3-kinase were identified at appropriate frequencies (Figure 3, Supplementary Figure 7). Twenty-nine genes related to RTK-RAS-MAPK, PI3K-AKT, MYC, mTOR, p53, FAS, FOXO1, and PTEN were selected (Figure 6A). Reconstituting the data matrix for CNVs revealed a bias for target molecules and signaling pathways in each PCNSL specimen (Figure 6A). Of these, a few somatic mutations were also identified in *ERBB3* (SNVs/INDELs: 22.2%, and CNVs: 25.9%), *ERBB2* (3.7% and 22.2%), *MYC* (7.4% and 11.1%), *RAF1* (7.4% and 33.3%), *TP53* (3.7% and 29.6%), *NRAS* (3.7% and 11.1%), *MAP2K2* (3.7% and 7.4%), and *MYCN* (3.7% and 7.4%) (Figures 2 and 6A, and Supplementary Figures 5 and 6A). Based on the CNVs with gains in genes related to PI3K-AKT, RTK-RAS-MAPK, MYC, mTOR as upregulated oncopathways, and CNVs with losses in genes related to PTEN, FAS, FOXO1, and p53 as downregulated proapoptotic pathways, the 27 PCNSL patients were divided into two subgroups as shown in Figure 6A and 6B. The subgroup associated with gains of CNVs for oncopathways and losses of CNVs for proapoptotic pathways showed poor prognoses compared to the other group (HR = 2.813, 95%CI: 1.047–7.427, P = 0.0406) (Figure 6B). Kaplan-Meier curves were also distinctly different (log-rank test: P = 0.0278) (Figure 6C). In detail, the subgroup associated with gains of CNVs for the oncopathway and losses of CNVs for the proapoptotic pathway showed a poor prognosis compared to that with gains only or losses only (HR = 3.488, P = 0.0254) (Supplementary Figure 14A and 14B). The subgroups associated with or without gains of CNVs for the oncopathway showed a small difference (HR = 1.407, P = 0.6394), whereas those with losses of CNVs for proapoptotic pathway showed distinct Kaplan-Meier curves (HR = 2.445, P = 0.0805) (Supplementary Figure 14A and 14B). These results suggest that a combined pathway derived from the RTK-RAS-MAPK oncopathway and PI3K-AKT proapoptotic pathway following stimulus-dependent activation of receptors is a promising molecular target for cancer therapies; these targets also include EGF receptor family members including EGFR, ERBB2/3/4, and MET and the FAS death receptor (Figure 6D).

## DISCUSSION

Here, we performed multiplex PCR amplification of the entire coding regions of 409 cancer-related genes and exome-sequencing on next-generation Ion Proton semiconductor sequencers for 27 PCNSL tumors and matched normal tissues. The most common nucleotide substitutions were C/G>T/A transitions accounting for 42.8%, which might be reflected in the APOBEC/AID deaminases [33–35]. The most frequent somatic mutations including SNVs and INDEL mutations were found in *PIM1, MYD88, CD79B, IRF4, MYH11, KMT2D, PAX5, DCC*, and *ERBB3*, and a few splice site mutations were found in *CD79B, PIM1*, and *PTCH1*. Further, Kaplan-Meier analysis showed poor and good prognoses in somatic mutations of *MYH11* and *PAX5* with decreased and increased copy numbers, respectively, in PCNSL patients. Furthermore, somatic mutations in MYH11 were located into the functional domain such as myosin large ATPase. Therefore, these two candidates, *MYH11* and *PAX5*, are the most significant diagnosis and prognostic markers for PCNSL tumorigenesis and patient survival. In addition, combined copy number alterations with amplifications in RTK-RAS-MAPK signaling and losses in the PTEN-PI3K-AKT proapoptotic pathway suggested poor prognoses in PCNSL. In summary, the study may enable the development of practical molecular targeted therapies, such as RTKs including EGF receptor family upstream at RAS-MAP-kinase and FAS death receptor merged into the PI3K-PTEN-AKT proapoptotic pathway based on the diagnosis and prognosis in PCNSL.

### Somatic mutation in PCNSL

Exome-sequencing analysis in PCNSL previously revealed that mutations occurred most frequently in *MYD88* (38%), *CD79B* (30%), *PIM1* (22%), and *TBL1XR1* (19%) [20]. In addition, PCNSL tumorigenesis contains moderately mutated genes including *ETV6* (16%), *IRF4* (14%), *IRF2BP2* (11%), and *EBF1* (11%). However, a recent exome-sequencing analysis revealed somatic hypermutations in *PIM1* (100%), *BTG2* (92.7%), and *MYD88* (79-85.4%), as well as focal deletions and somatic mutations in the HLA genes which were linked to poor prognoses in PCNSL [36, 37]. Our study also demonstrated that *PIM1* (85.1%), *MYD88* (81.4%), *CD79B* (59.2%) contained somatic mutations (Figure 2A). Thus, the mutations in PIM1, MYD88, and CD79B were observed in a larger population in PCNSL, although mutation frequencies were greatly affected by the sample numbers and their biases. In contrast, a median of 22.2% of SNVs overlapped with the RGYW motif targeted by somatic hypermutation, and a median of 7.9% affected its hotspot position, which is considered a significant somatic hypermutation within PCNSL pathogenesis, in addition to minor gene alterations in *CSMD2/3*, *ODZ4*, and *PTPRD* [21]. In this study, missense mutations were detected in *CSMD3* (18.5%) (Figure 2A) but did not show a significant HR compared to the wild-type gene (Table 1 and Figure 4). Besides, the genome-wide analysis has reported that several genes recurrently affected in PCNSL are common with systemic DLBCL, including loss of TNFAIP3, PRDM1, GNA13, TMEM30A, TBL1XR1, B2M, CD58, activating mutations of CD79B, CARD11, and translocations IgH-BCL6 [37]. Furthermore, the integrated analysis has demonstrated enrichment of pathways associated with immune response, proliferation, apoptosis, and lymphocyte differentiation [37]. Our results in the study are also consistent with these data in part, such as integrated pathways associated with RTK-RAS-MAPK oncopathway and PI3K-PTEN-AKT proapoptotic pathway. While, whole-exome sequencing and gene expression profiling have verified the importance of NF-ĸB deregulation in primary mediastinal B-cell lymphoma (PMBL). NF-ĸB inhibitor epsilon (NFKBIE) aberrations are common genetic event across B-cell malignancies, and frequent NFKBIE deletions are associated with poor prognosis in PMBL [38].

On the other hand, some of somatic mutations were detected by low frequencies (e.g. ADAMTS20, EPHA3, and TRRAP), which should be validated to be the reliable mutations. Then, we performed PolyPhen-2 and SIFT analyses for the mutational significances in the representative genes including EPHA3, ADAMTS20, and TRRAP (Supplementary Table 2). The results from allele frequencies >15% showed relatively low SIFT scores and high PolyPhen-2 scores in EPHA3, ADAMTS20, and TRRAP, while the mutations with allele frequencies <15% showed relatively weak significances. These data suggest that even the Ion Torrent Next-Generation Sequencing and Ion Torrent Server are difficult to detect minor alleles in significant mutations. The Ion Torrent Server built-in-modules as variantCaller and Ion Reporter Software/Server have been optimized for the Ion Reporter semiconductor sequencer, and generally used as described [39]. The recent study has also demonstrated that the Ion Reporter showed the best performance among the individual callers including Ion Reporter, Poor man’s, MuTect, and Varscan [40]. On the other hand, a few studies by Ion Torrent NGS have been reanalyzed by a different variant calling tool/pipeline, Broad's Genome Analysis Toolkit (GATK) [41]. Thus, reanalysis by the GATK or a comparison of SNV allele frequencies to a larger population cohort (e.g. Tohoku Medical Megabank Project) might be effective to detect minor gene alterations. Also, the threshold of Ion Torrent Software/Server should be preset to be more sensitive for a low frequency.

### Chromosomal imbalances and oncogenic alleles associated with high risks in PCNSL

Recurrent CNVs have been observed in frequent chromosome losses affecting 6p21.32 (79%) corresponding to the HLA locus, 6q16.3 (37%), 6q21.1-q25 (34%), and 6q14.1-q16.3 (27%) on chromosome 6, and 12q12-q22 (27%), 7q21.11-q21.12 (20%), and 9p21 regions in PCNSL [13, 14, 27, 42]. In contrast, 7q31.1-q31.2 showed a chromosome gain [14], and a recent study reported that copy number amplifications at 7q35 shorten progression-free survival of patients with PCNSL [36]. However, in this study, most CNVs in chromosomal regions appeared to be increased rather than decreased (Supplementary Figure 15). Chromosomal imbalances were identified in chromosome 6 as a loss and chromosomes 1, 3, 7, and 12 as gains (Figure 5A). Except for AR, the candidate markers from somatic mutations were also detected by CNVs (Figure 5C). Further, differential CNVs in genes including *EPHA3*, *KMT2A, PAX5*, and *RUNX1* clearly divided the Kaplan-Meier curves of the two subgroups with or without CNVs in cases with poor prognosis (Figure 5E, Supplementary Figure 13A and 13B). Our data and analyses are limited and incomplete because of the small number of PCNSL specimens, and thus optimized combinations of these candidate markers for genome diagnostics in PCNSL should be further examined.

### Oncosignaling and therapeutic strategies in PCNSL

The B-cell receptor (BCR)/Toll-like receptor (TLR)-NF-ĸB pathways are altered in >90% of PCNSL [42]. Mutations affecting BCR, TLR, and NF-κB signaling are involved in chromatin structure and modification, cell cycle, and immune recognition [20, 21]. Such immune response signaling pathways are well-known in PCNSL within DLBCL. This study also identified immune disease-related genes associated with somatic mutations such as *MYD88* and *BCL10* in NF-κB signaling, *CD79B, PAX5*, and *BCR* in B-cell development, and *MYH11, RUNX1*, and *TET2* in leukemia (Figure 3, Supplementary Figure 7). Additionally, *PIM1* and *MARK1* in Ser/Thr-kinases, *ERBB3, FLT4*, and *PDGFRA/B* in RTKs and *EPHA3/7* in non-RTKs were identified in stimulus-dependent intracellular signaling pathways (Figure 3). Of these, somatic mutations in *EPHA3* (HR = 3.54 × 10^11^, P = 0.0033) and *MYH11* (HR = 11.94, P = 0.0043) were associated with short survival, while *PAX5* mutations were associated with long survival (HR=0.05, P=0.0307). Additionally, CNVs in regions including *EPHA3* (3p11.1) and *MYH11* (16p13.11) were relatively decreased (37.5%, respectively), whereas that of *PAX5* (9p13.2) was relatively increased (83.3%) (Figure 5A and 5C, Supplementary Figure 12). Further, the provisional combination of CNVs composed of *EPHA3*, *MYH11*, and *PAX5* demonstrated effects on their survival in terms of the hazard ratios (Figure 5E, Supplementary Figure 13A and 13B).

It is known that the oncogenic mutation in growth factor receptor-bound protein 2 (GRB2), which mediates growth factors and RAS-MAPK signaling, governs MEK1/2 activities downstream at MAP-kinase in DLBCL-type PCNSL [36]. We also focused on the RTK-RAS-MAPK oncopathway and PI3K-PTEN-AKT proapoptotic pathway to determine copy number alterations (Figure 6A). Interestingly, reciprocal copy number alterations of the loss of copy numbers related to PI3K-PTEN-AKT divided the Kaplan-Meier curves, while a gain of copy numbers related to RTK-RAS-MAPK did not divide the two subgroups (Supplementary Figure 14A and 14B). However, the merged copy number alterations clearly divided the survival curves for the two subgroups (Figure 6C, Supplementary Figure 14A and 14B). Therefore, we considered that sequential copy number alterations of both the RTK-RAS-MAPK and PI3K-PTEN-AKT pathways and combined signaling activation and deregulation derived from the two pathways might enhance PCNSL tumorigenicity (Figure 6D). In addition, somatic mutations in genes related to both pathways were identified, e.g., *ERBB2/3/4*, *MYC, RAF1, TP53, PIK3CD, NRAS*, and *MAP2K2* (also known as *MEK2*) (Figure 3, Supplementary Figure 7). Thus, receptor tyrosine kinases including the EGF receptor family upstream of RAS-MAP-kinase and FAS death receptor merged into the PI3K-PTEN-AKT proapoptotic pathway could be promising therapeutic targets for genome diagnostics and as prognostic markers in PCNSL. Here, we only showed the results based on the Ion Torrent NGS data, whereas did not demonstrate directly the evidences for the BCR/TLR pathway including MYH11, PAX5, RUNX1 to RAS-MAPK or PI3K-PTEN-AKT, and the genetic interaction between the BCR/TLR pathway and RTK in molecular biology, biochemistry, and biophysics. However, the proposal would provide a hint for the further investigation of above-described genetic and molecular interactions as promising targets for the PCNSL treatments.

## MATERIALS AND METHODS

### Clinical specimens

Twenty-seven patients with PCNSL were diagnosed and treated at Chiba University, Toyama Prefectural Central Hospital, Wakayama Medical University School of Medicine, and Yamaguchi University. Clinical characteristics are shown in Supplementary Figure 9. The study was approved by all institutional ethics committees.

### Semiconductor-based next-generation sequencing

DNA was extracted from the frozen and formalin-fixed paraffin-embedded (FFPE) tissues and peripheral blood using a QIAamp DNA Mini Kit (Qiagen, Hilden, Germany) and QIAamp DNA FFPE Tissue Kit (Qiagen) according to the manufacturer's instructions. The TaqMan RNase P Detection Reagents Kit (Thermo Fisher Scientific, Waltham, MA, USA) was used to quantify the purified DNAs. Semiconductor-based next-generation sequencing was performed as described previously [39]. DNA was used for multiplex PCR amplification with an Ion Ampliseq Comprehensive Cancer Panel (Thermo Fisher Scientific), enabling targeted coverage of all exons of 409 cancer-related genes (covered regions: 97.7% of total). The templates were sequenced after emulsion PCR with an Ion PI chip using the Ion PI HI-Q Chef Kit (Thermo Fisher Scientific). The obtained 30,860 amplicons represented more than 124.7 million target sequences in the total specimens.

### Identification of SNVs and INDELs

Genome Reference Consortium Human Build 37 (GRCh37/hg19) was used as a reference. Alignment to the GRCh37/hg19 genome and sequencing read counting were performed in Torrent Suite version 5.0 (Thermo Fisher Scientific). Somatic mutations including SNVs and INDELs were detected using statistics in tumor and matched normal control samples from the Ion Reporter software 5.0 tumor-normal workflow (Thermo Fisher Scientific) or the provided control sequence data (Thermo Fisher Scientific), as described previously [39]. The 20× sequencing coverage and variant frequency >15% of the total number of distinct tags were used after the cutoff. Mutations were called if they occurred in <0.1% of reads in the normal control (minor allele frequency) and were absent from dbSNP as well as the 1000 Genomes Project database. Integrative Genomics Viewer (IGV) software ( http://www.broadinstitute.org/igv) was used to filter out possible strand-specific errors, such as a mutation that was only detected in the forward or reverse DNA strand but not in both strands.

### CNV detection

CNVs were detected by Ion Reporter software (Thermo Fisher Scientific) using an algorithm based on a hidden Markov model. Recurrent genomic regions with CNVs were identified by copy numbers ≥3 (gain) and <2 (loss). CNVs were analyzed for somatic mutations, signaling pathways, and patient's prognoses.

### Sanger sequencing

Target sequences were surveyed by UCSC Genome Browser on Human GRCh37/hg19 Assembly ( https://genome.ucsc.edu/). Sanger sequencing is performed according to standard protocols. In brief, target DNAs were amplified from total DNAs with primers (Supplementary Figure 8A) by polymerase chain reaction (PCR), followed by Big Dye Terminator V3.1 reaction (Thermo Fisher Scientific). Sequences were detected and visualized by Applied Biosystems 3130xl Genetic Analyzer.

### Gene annotation

Functional gene annotation was performed using GOstat ( http://gostat.wehi.edu.au/) and The Database for Annotation, Visualization and Integrated Discovery (DAVID) v6.8 ( https://david.ncifcrf.gov/) as described [43].

### Clustering analysis

Somatic mutations and CNVs derived from 27 PCNSL specimens were clustered according to the mutation profiling per specimen using JMP built-in modules (SAS Institute, Inc., Tokyo, Japan) as described [43].

### Kaplan-Meier analysis

The Kaplan-Meier method was used to estimate survival distributions for each group with the log-rank test among subgroups using JMP built-in modules (SAS Institute Inc.) [43]. Hazard ratios (HR) and 95% confidence intervals (CI) were calculated based on a logistic regression model with respect to clinical variables that were assessed by multivariate analysis with stepwise selection to compare groups. Overall survival (OS) was defined as the date of diagnosis of PCNSL to the date of death or last follow-up.

### Statistics

Statistical analyses were performed using JMP v10 (SAS Institute Inc.). Statistical significance was assessed using a log-rank test. P < 0.05 was considered statistically significant.

## SUPPLEMENTARY MATERIALS FIGURES AND TABLES




