# Supplementary material for: Target amplicon exome-sequencing identifies promising diagnosis and prognostic markers involved in RTK-RAS and PI3K-AKT signaling as central oncopathways in primary central nervous system lymphoma
Source: Oncotarget. 2018 Jun 8;9(44):27471–86. doi: 10.18632/oncotarget.25463 (PMC6007945; doi:10.18632/oncotarget.25463)
Supplement: Supplementary file 1 [file oncotarget-09-27471-s001.pdf]

Target amplicon exome-sequencing identifies promising diagnosis and prognostic markers involved in RTK-RAS and PI3K-AKT signaling as central oncopathways in primary central nervous system lymphoma

SUPPLEMENTARY MATERIALS

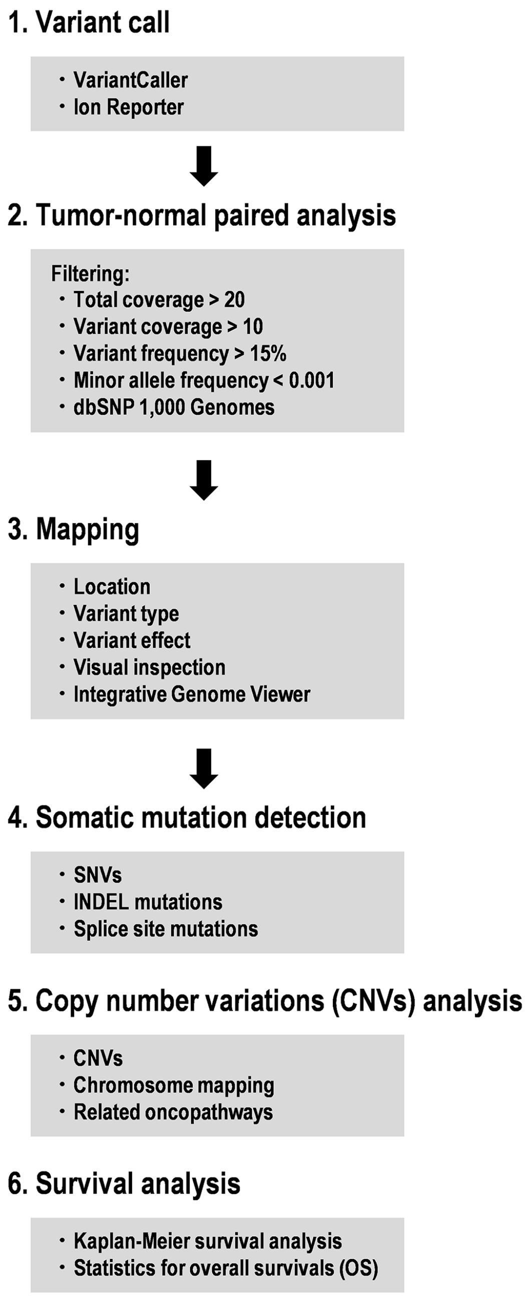

Supplementary Figure 1: Schematic representation of data analysis. Workflow to identify somatic single-nucleotide variations (SNVs), insertion and deletion mutations (INDELs), and copy number variations (CNVs) from the sequencing data.

ABL1	CBL*	EP300*	GATA2	LAMP1	MYH11*	PLAG1	SMARCA4	WHSC1*
ABL2*	CCND1*	EP400	GATA3	LCK	MYH9	PLCG1*	SMARCB1	WRN
ACVR2A	CCND2*	EPHA3*	GDNF	LIFR*	NBN	PLEKHG5*	SMO*	WT1
ADAMTS20*	CCNE1	EPHA7*	GNA11	LPHN3*	NCOA1	PML	SMUG1	XPA
AFF1*	CD79A	EPHB1	GNAQ	LPP*	NCOA2	PMS1	SOCS1*	XPC*
AFF3	CD79B*	EPHB4	GNAS*	LRP1B*	NCOA4	PMS2	SOX11*	XPO1
AKAP9*	CDC73	EPHB6*	GPR124*	LTF	NF1	POT1	SOX2	XRCC2
AKT1	CDH1	ERBB2*	GRM8	LTK	NF2	POU5F1	SRC	ZNF384
AKT2	CDH11	ERBB3*	GUCY1A2*	MAF	NFE2L2*	PPARG	SSX1	ZNF521
AKT3	CDH2	ERBB4	HCAR1	MAFB	NFKB1	PPP2R1A	STK11	
ALK*	CDH20*	ERCC1	HIF1A	MAGEA1	NFKB2*	PRDM1*	STK36*	
APC*	CDH5	ERCC2	HLF	MAGI1*	NIN	PRKAR1A	SUFU	
AR*	CDK12	ERCC3	HNF1A	MALT1	NKX2-1	PRKDC*	SYK	
ARID1A*	CDK4	ERCC4	HOOK3	MAML2	NLRP1*	PSIP1	SYNE1*	
ARID2	CDK6*	ERCC5	HRAS	MAP2K1	NOTCH1	PTCH1	TAF1	
ARNT	CDK8	ERG*	HSP90AA1	MAP2K2*	NOTCH2	PTEN	TAF1L*	
ASXL1*	CDKN2A	ESR1*	HSP90AB1	MAP2K4	NOTCH4	PTGS2*	TAL1	
ATF1	CDKN2B	ETS1*	ICK*	MAP3K7	NPM1	PTPN11	TBX22	
ATM	CDKN2C	ETV1	IDH1	MAPK1*	NRAS*	PTPRD*	TCF12*	
ATR*	CEBPA*	ETV4	IDH2	MAPK8	NSD1*	PTPRT	TCF3*	
ATRX*	CHEK1	EXT1*	IGF1R	MARK1	NTRK1*	RAD50	TCF7L1	
AURKA	CHEK2	EXT2	IGF2	MARK4	NTRK3*	RAF1*	TCF7L2	
AURKB	CIC*	EZH2	IGF2R*	MBD1*	NUMA1*	RALGDS*	TCL1A*	
AURKC	CKS1B	FAM123B	IKBKB	MCL1	NUP214	RARA	TET1	
AXL	CMPK1	FANCA*	IKBKE	MDM2	NUP98	RB1	TET2*	
BAI3*	COL1A1	FANCC	IKZF1	MDM4	PAK3	RECQL4	TFE3	
BAP1	CRBN	FANCD2	IL2	MEN1	PALB2	REL	TGFBR2	
BCL10	CREB1	FANCF	IL21R*	MET	PARP1	RET*	TGM7*	
BCL11A	CREBBP*	FANCG	IL6ST*	MITF	PAX3	RHOH	THBS1*	
BCL11B	CRKL	FAS	IL7R	MLH1*	PAX5*	RNASEL	TIMP3	
BCL2*	CRTC1*	FBXW7	ING4	MLL	PAX7*	RNF2	TLR4*	
BCL2L1	CSF1R	FGFR1	IRF4*	MLL2	PAX8	RNF213*	TLX1	
BCL2L2	CSMD3*	FGFR2*	IRS2	MLL3	PBRM1	ROS1*	TNFAIP3*	
BCL3*	CTNNA1	FGFR3	ITGA10	MLLT10	PBX1*	RPS6KA2	TNFRSF14	
BCL6*	CTNNB1*	FGFR4	ITGA9	MMP2	PDE4DIP	RRM1	TNK2*	
BCL9	CYLD	FH	ITGB2	MN1	PDGFB	RUNX1*	TOP1*	
BCR*	CYP2C19	FLCN	ITGB3	MPL	PDGFRA*	RUNX1T1	TP53*	
BIRC2*	CYP2D6	FLI1	JAK1*	MRE11A	PDGFRB*	SAMD9*	TPR*	
BIRC3	DAXX	FLT1	JAK2	MSH2	PER1	SBDS	TRIM24	
BIRC5	DCC*	FLT3	JAK3	MSH6	PGAP3*	SDHA	TRIM33*	
BLM	DDB2	FLT4*	JUN	MTOR	PHOX2B	SDHB	TRIP11	
BLNK*	DDIT3	FN1	KAT6A*	MTR	PIK3C2B	SDHC	TRRAP*	
BMPR1A	DDR2*	FOXL2	KAT6B	MTRR	PIK3CA	SDHD	TSC1	
BRAF*	DEK	FOXO1*	KDM5C	MUC1	PIK3CB	SEPT9	TSC2	
BRD3	DICER1*	FOXO3	KDM6A	MUTYH*	PIK3CD	SETD2*	TSHR	
BRIP1	DNMT3A	FOXP1*	KDR	MYB	PIK3CG	SF3B1	UBR5*	
BTK	DPYD*	FOXP4*	KEAP1	MYC*	PIK3R1	SGK1	UGT1A1	
BUB1B	DST*	FZR1	KIT*	MYCL1	PIK3R2	SH2D1A	USP9X*	
CARD11*	EGFR	G6PD	KLF6	MYCN*	PIM1*	SMAD2	VHL	
CASC5	EML4	GATA1	KRAS	MYD88*	PKHD1*	SMAD4	WAS	

NOTE: Asterisks (*) indicate the genes detected in the study

Supplementary Figure 2: Gene list of Ion Ampliseq Comprehensive Cancer Panel including 409 cancer-related genes. A total of 136 genes (33.25%) was detected in the 27 PCNSL specimens in the study (variant frequency > 15%). Asterisks (*) indicate the genes detected in this study.

Sample No.	Types	Mapped reads	Reads on target %	Coverage depth	Uniformity of base coverage	Coverage 1x	Coverage 20x	Coverage 100x	Coverage 500x
1	PCNSL tumors	7,121,997	97.3	449.2	95.2	99.8	99.0	94.3	38.9
2	PCNSL tumors	7,200,140	95.3	427.5	93.5	99.8	98.0	92.0	31.6
3	PCNSL tumors	6,363,766	97.6	398.6	94.8	99.8	98.6	92.6	27.2
4	PCNSL tumors	4,126,465	96.5	258.7	95.4	99.7	98.0	87.4	8.8
5	PCNSL tumors	3,403,125	98.3	215.4	94.4	99.4	97.0	84.4	2.9
6	PCNSL tumors	4,342,607	97.7	263.3	94.7	99.7	97.6	87.0	9.0
7	PCNSL tumors	5,712,861	97.9	358.4	96.6	99.8	98.6	94.9	14.3
8	PCNSL tumors	3,704,279	98.0	233.3	95.0	99.5	97.4	86.2	4.1
9	PCNSL tumors	7,531,321	98.1	467.9	95.5	99.8	98.8	95.1	37.2
10	PCNSL tumors	3,615,471	96.8	220.0	93.2	99.7	96.9	81.3	4.0
11	PCNSL tumors	6,247,085	97.3	391.5	96.2	99.8	98.7	94.8	25.2
12	PCNSL tumors	3,813,972	98.2	232.5	82.6	99.1	91.8	64.8	12.2
13	PCNSL tumors	4,372,564	98.5	272.7	90.4	99.4	95.7	83.1	10.0
14	PCNSL tumors	4,231,742	97.9	256.6	90.0	99.3	95.5	80.7	9.1
15	PCNSL tumors	6,778,234	97.6	428.0	96.2	99.8	98.8	95.4	31.0
16	PCNSL tumors	4,025,504	98.0	251.0	95.4	99.7	98.1	88.2	4.7
17	PCNSL tumors	4,105,783	97.8	246.4	86.5	99.4	93.7	74.0	10.4
18	PCNSL tumors	3,181,222	97.9	198.0	94.7	99.6	97.3	74.5	4.3
19	PCNSL tumors	3,670,203	98.6	235.4	95.0	99.6	97.3	87.4	4.6
20	PCNSL tumors	3,466,785	98.3	220.3	94.8	99.6	97.4	84.8	3.0
21	PCNSL tumors	5,715,054	98.7	366.7	94.9	99.6	97.9	92.7	21.5
22	PCNSL tumors	2,437,808	96.9	145.0	88.4	98.9	91.7	55.1	2.3
23	PCNSL tumors	3,175,593	94.6	189.3	89.2	99.2	94.2	67.3	5.3
24	PCNSL tumors	4,130,378	94.9	240.7	83.7	99.3	92.4	70.1	11.6
25	PCNSL tumors	5,277,734	98.3	336.9	95.4	99.6	98.0	93.0	15.5
26	PCNSL tumors	3,922,285	98.1	248.6	94.1	99.5	96.9	87.2	4.4
27	PCNSL tumors	3,094,304	97.6	182.6	85.9	99.2	91.9	62.9	5.4
1N	Matched normal controls	6,767,427	98.9	447.0	93.9	99.7	97.3	93.3	36.0
2N	Matched normal controls	6,907,124	98.8	455.6	93.5	99.7	97.1	93.0	38.0
3N	Matched normal controls	7,643,458	99.1	507.6	93.1	99.7	97.4	93.2	43.8
4N	Matched normal controls	4,449,899	98.4	255.8	80.8	99.0	92.4	65.6	11.1
5N	Matched normal controls	3,361,991	98.7	221.9	92.7	99.5	95.7	78.6	6.3
6N	Matched normal controls	4,432,024	98.9	292.8	93.9	99.6	96.7	90.1	10.1
7N	Matched normal controls	7,048,280	99.1	468.0	92.8	99.7	97.1	92.5	38.8
8N	Matched normal controls	6,801,999	99.0	450.7	93.2	99.6	97.0	92.7	35.9
9N	Matched normal controls	1,649,377	92.4	100.9	93.1	99.0	95.2	46.3	0.0
10N	Matched normal controls	5,276,443	98.9	350.2	93.9	99.6	96.8	92.1	18.1
11N	Matched normal controls	6,500,675	98.9	432.2	93.5	99.7	97.2	92.8	33.6
12N	Matched normal controls	6,209,276	99.0	412.1	93.0	99.7	96.9	91.8	30.1
13N	Matched normal controls	4,055,598	96.8	251.1	91.8	99.0	95.4	84.6	6.7
14N	Matched normal controls	2,451,487	96.5	153.0	97.1	99.6	97.8	84.0	0.1
15N	Matched normal controls	2,482,097	96.6	158.3	94.0	99.3	95.5	75.4	0.3
16N	Matched normal controls	6,920,277	98.9	458.1	93.5	99.7	97.0	93.1	39.7
17N	Matched normal controls	6,998,024	98.6	463.6	93.2	99.7	96.8	92.9	39.1

**Tumors**	**Average**	**4,621,047**	**97.5**	**286.5**	**92.7**	**99.5**	**96.6**	**83.4**	**13.3**
**(N = 27)**	**Max**	**7,531,321**	**98.7**	**467.9**	**96.6**	**99.8**	**99.0**	**95.4**	**38.9**
	**Min**	**2,437,808**	**94.6**	**145.0**	**82.6**	**98.9**	**91.7**	**55.1**	**2.3**

**Controls**	**Average**	**5,291,497**	**98.1**	**345.8**	**92.8**	**99.5**	**96.4**	**85.4**	**22.8**
**(N = 17)**	**Max**	**7,643,458**	**99.1**	**507.6**	**97.1**	**99.7**	**97.8**	**93.3**	**43.8**
	**Min**	**1,649,377**	**92.4**	**100.9**	**80.8**	**99.0**	**92.4**	**46.3**	**0.0**

**Total**	**Average**	**4,880,085**	**97.7**	**309.4**	**92.7**	**99.5**	**96.5**	**84.2**	**17.0**
**(N = 44)**	**Max**	**7,643,458**	**99.1**	**507.6**	**97.1**	**99.8**	**99.0**	**95.4**	**43.8**
	**Min**	**1,649,377**	**92.4**	**100.9**	**80.8**	**98.9**	**91.7**	**46.3**	**0.0**
			**(a)**	**(b)**			**(c)**		

Supplementary Figure 3: Summary of targeted amplicon sequencing data for PCNSL specimens. In this study, 27 PCNSL tumors and 18 matched normal controls were sequenced. The percent (%) reads of targets (**a**) in tumors, matched paired normal tissues, and total specimens were 97.5 (94.6–98.7), 98.1 (92.4–99.1), and 97.7 (92.4–99.1) and coverage depths (**b**) were 286.5 (145.0–467.9), 345.8 (100.9–507.6), and 309.4 (100.9–507.6), respectively. The percent (%) 20x coverage in total specimens (**c**) was 96.5 (91.7–99.0).

A

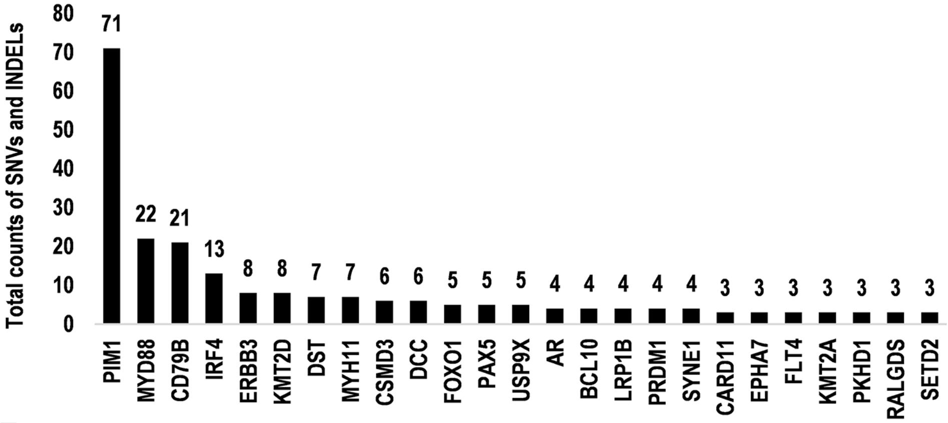

B

Symbol	Refseq	Chromosome band	Alias	Description	Total counts
PIM1	NM_001243186	6p21.2	PIM1, PIM, Pim-1 proto-oncogene, serine/threonine kinase	Proto-oncogene serine/threonine-protein kinase	71
MYD88	NM_002648	3p22.2	MYD88, MYD88D, myeloid differentiation primary response 88	Myeloid differentiation primary response 88	22
CD79B	NM_000626	17q23.3	CD79B, AGM6, B29, IGB, CD79b molecule	CD79b molecule, immunoglobulin-associated beta	21
IRF4	NM_001195286	6p25.3	IRF4, LSIRF, MUM1, NF-EM5, SHEP8, interferon regulatory factor 4	Interferon regulatory factor 4	13
ERBB3	NM_001982	12q13.2	ERBB3, ErbB-3, HER3, LCCS2, MDA-BF-1, c-erbB-3, c-erbB3, erbB3-S, p180-ErbB3, p45-sErbB3, p85-sErbB3, erb-b2 receptor tyrosine kinase 3	Receptor tyrosine-protein kinase erbB-3, also known as HER3 (human epidermal growth factor receptor 3)	8
KMT2D	NM_003482	12q13.12	KMT2D, ALR, KABUK1, MLL2, MLL4, lysine methyltransferase 2D, histone-lysine methyltransferase 2D, TNRC21, AAD10, KMS, CAGL114	Histone-lysine N-methyltransferase 2D	8
DST	NM_001144769	6p12.1	DST, BP240, BPA, BPAG1, CATX-15, CATX15, D6S1101, DMH, DT, EBSB2, HSAN6, MACF2, dystonin	Dystonin (DST), also known as Bullous pemphigoid antigen 1,(BPAG1)	7
MYH11	NM_001040113	16p13.11	MYH11, AAT4, FAA4, SMHC, SMMHC, myosin, heavy chain 11, smooth muscle, myosin heavy chain 11	Myosin-11	7
CSMD3	NM_198123	8q23.3	CUB And Sushi Multiple Domains 3, CUB And Sushi Multiple Domains Protein 3, KIAA1894	CUB and Sushi multiple domains 3	6
DCC	NM_005215	18q21.2	DCC, DCC netrin 1 receptor, CRC18, CRCR1, IGDCC1, MRMV1, NTN1R1, HGPPS2	Deleted in Colorectal Carcinoma	6
FOXO1	NM_002015	13q14.11	FOXO1, FKH1, FKHR, FOXO1A, forkhead box O1	Forkhead box protein O1 (FOXO1), also known as forkhead in rhabdomyosarcoma	5
PAX5	NM_001280547	9p13.2	PAX5, ALL3, BSAP, paired box 5	Paired box protein Pax-5	5
USP9X	NM_001039590	Xp11.4	USP9X, DFFRX, FAF, FAM, MRX99, MRXS99F, ubiquitin specific peptidase 9, X-linked	Probable ubiquitin carboxyl-terminal hydrolase FAF-X	5
AR	NM_001011645	Xq12	AR, AIS, AR8, DHTR, HUMARA, HYSP1, KD, NR3C4, SBMA, SMAX1, TFM, androgen receptor	Androgen receptor (AR), also known as NR3C4 (nuclear receptor subfamily 3, group C, member 4)	4
BCL10	NM_003921	1p22.3	BCL10, CARMEN, CIPER, CLAP, c-E10, mE10, IMD37, B-cell CLL/lymphoma 10	B-cell lymphoma/leukemia 10	4
LRP1B	NM_018557	2q22.1-q22.2	LRP1B, LRP-DIT, LRPDIT, LDL receptor related protein 1B, LRP-1B	Low-density lipoprotein receptor-related protein 1B	4
PRDM1	NM_001198	6q21	PRDM1, BLIMP1, PRDI-BF1, PR domain 1, PR/SET domain 1	PR domain zinc finger protein 1 also known as BLIMP-1	4
SYNE1	NM_001099267	6q25.2	SYNE1, 8B, ARCA1, C6orf98, CPG2, EDMD4, MYNE1, Nesp1, SCAR8, dJ45H2.2, spectrin repeat containing nuclear envelope protein 1, KASH1	Enaptin also known as nesprin-1 or synaptic nuclear envelope protein 1 (syne-1)	4
CARD11	NM_032415	7p22.2	CARD11, BENTA, BIMP3, CARMA1, IMD11, PPBL, caspase recruitment domain family member 11, IMD11A	Caspase recruitment domain-containing protein 11	3
EPHA7	NM_001288629	6q16.1	EPHA7, EHK-3, EHK3, EK11, HEK11, EPH receptor A7	Ephrin type-A receptor 7	3
FLT4	NM_002020	5q35.3	FLT4, FLT41, LMPH1A, PCL, VEGFR3, FLT-4, VEGFR-3, fms related tyrosine kinase 4	Fms-related tyrosine kinase 4, also known as FLT4	3
KMT2A	NM_001197104	11q23.3	KMT2A, ALL-1, CXXC7, HRX, HTRX1, MLL, MLL/GAS7, MLL1, MLL1A, TET1-MLL, TRX1, WDSTS, MLL-AF9, lysine methyltransferase 2A, Histone-lysine N-methyltransferase HRX	Histone-lysine N-methyltransferase 2A also known as acute lymphoblastic leukemia 1 (ALL-1), myeloid/lymphoid or mixed-lineage leukemia 1 (MLL1), zinc finger protein HRX (HRX)	3
PKHD1	NM_138694	6p21.2-p12	Fibrocystin, TIGM1, FCYT, ARPKD, PKD4, FPC	Fibrocystin	3
RALGDS	NM_006266	9q34.13-q34.2	RALGDS, RGDS, RGF, RalGEF, ral guanine nucleotide dissociation stimulator	Ral guanine nucleotide dissociation stimulator	3
SETD2	NM_012271	3p21.31	SETD2, HBP231, HIF-1, HIP-1, HYPB, KMT3A, SET2, p231HBP, HSPC069, LLS, SET domain containing 2	SET domain containing 2	3

Supplementary Figure 4: Total numbers of SNVs and INDELs counted in each gene. (A) Representative genes with the number of somatic mutations including of SNVs and INDELs (N ≥ 3). **(B)** Description of the genes shown in A.

Symbol	Type	Sample 20	Sample 25	Sample 19	Sample 14	Sample 13	Sample 23	Sample 24	Sample 27	Sample 21	Sample 16	Sample 15	Sample 3	Sample 4	Sample 18	Sample 26	Sample 8	Sample 10	Sample 9	Sample 22	Sample 5	Sample 11	Sample 1	Sample 12	Sample 17	Sample 6	Sample 7	Sample 2	(N)
ABL2	SNV																					1							1
AFF1	SNV	1																											1
AKAP9	SNV	1																											1
ALK	SNV															1													1
APC	SNV		1																										1
ARID1A	SNV										1																		1
ASXL1	SNV		1																										1
ATR	SNV				1																								1
ATRX	SNV													1															1
BAI3	SNV																	1											1
BCL2	SNV					2																							1
BCL3	SNV												1																1
BCL6	SNV																										1		1
BIRC2	SNV																											1	1
BLNK	SNV						1																						1
BRAF	SNV												3																1
CBL	SNV									1																			1
CCND2	SNV								1																				1
CDH20	SNV													1															1
CDK6	SNV	1																											1
CEBPA	SNV					1																							1
CRTC1	SNV													1															1
CTNNB1	SNV																1												1
DDR2	SNV																1												1
DICER1	SNV		1																										1
EPHB6	SNV	1																											1
ERBB2	SNV											1																	1
ERG	SNV						1																						1
ESR1	SNV										1																		1
EXT1	SNV	1																											1
FANCA	SNV														1														1
FGFR2	SNV																							1					1
FOXP1	SNV																								1				1
GNAS	SNV											1																	1
GUCY1A2	SNV																		1										1
ICK	SNV																						1						1
IGF2R	SNV			1																									1
IL21R	SNV						1																						1
IL6ST	SNV										1																		1
JAK1	SNV									1																			1
KAT6A	SNV						1																						1
KIT	SNV			1																									1
KMT2C	SNV			1																									1
LIFR	SNV											1																	1
LPHN3	SNV																				1								1
LPP	SNV	1																											1
MAGI1	SNV																									1			1
MBD1	SNV				2																								1
MUTYH	SNV																								1				1
MYCN	SNV																						1						1
NFE2L2	SNV																							1					1
NFKB2	SNV								1																				1
NRAS	SNV																					1							1
NUMA1	SNV		1																										1
PAX7	SNV				1																								1
PBX1	SNV	1																											1
PGAP3	SNV																		1										1
PLEKHG5	SNV			1																									1
PRKDC	SNV															1													1
PTGS2	SNV				1																								1
PTPRD	SNV																				1								1
RET	SNV									1																			1
ROS1	SNV				1																								1
SMO	SNV							1																					1
SOCS1	SNV					1																							1
SOX11	SNV			1																									1
TAF1L	SNV																			1									1
TCF12	SNV								1																				1
TCF3	SNV		1																										1
TGM7	SNV							1																					1
THBS1	SNV															1													1
TLR4	SNV																			1									1
TNFAIP3	SNV																	1											1
TNK2	SNV														1														1
TOP1	SNV		1																										1
TP53	SNV	1																											1
TPR	SNV							1																					1
TRIM33	SNV					1																							1
WHSC1	SNV												1																1
XPC	SNV														1														1

1	missense
2	nonsense
3	stoploss

Supplementary Figure 5: Summary of single-nucleotide variations (SNVs) in the 27 PCNSL specimens. SNVs detected in PCNSL specimens (N = 1). Mutation types were shown in the data matrix including missense, nonsense, and stop loss. The numbers on the right side of panels (N) indicate the numbers of specimens with SNVs and/or INDELs, as well as splice site mutations.

A INDEL mutations

		Sample 20	Sample 24	Sample 27	Sample 21	Sample 16	Sample 8	Sample 10	Sample 6	Sample 25	Sample 19	Sample 14	Sample 13	Sample 23	Sample 15	Sample 3	Sample 4	Sample 18	Sample 26	Sample 9	Sample 22	Sample 5	Sample 11	Sample 1	Sample 12	Sample 17	Sample 7	Sample 2	(N)
CCND1	INDEL				7																								1
CREBBP	INDEL	4																											1
ETS1	INDEL						4																						1
GPR124	INDEL			4																									1
MAP2K2	INDEL					4																							1
NLRP1	INDEL								4																				1
SAMD9	INDEL		4																										1
STK36	INDEL							4																					1

4	INDEL/frameshiftDeletion
5	INDEL/frameshiftInsertion
6	INDEL/nonframeshiftDeletion
7	INDEL/nonframeshiftInsertion

B Splice site mutation

Symbol	Type	Sample 18	Sample 7	Sample 19	Sample 20	Sample 21	Sample 22	Sample 23	Sample 8	Sample 24	Sample 25	Sample 26	Sample 9	Sample 27	Sample 5	Sample 1	Sample 2	Sample 3	Sample 4	Sample 13	Sample 15	Sample 14	Sample 17	Sample 16	Sample 10	Sample 11	Sample 12	Sample 6	(N)
APC	SNV																		8										1
ASXL1	SNV																		8										1
KMT2D	SNV	8																											1
MYC	SNV																8												1
MYH9	INDEL																				8								1
NRAS	SNV																		8										1
NUP98	SNV																				8								1
PRDM1	SNV								8																				1
RARA	SNV												8																1
TPR	SNV																		8										1
USP9X	SNV																						8						1

8	Splice site

Supplementary Figure 6: Summary of insertion and deletion mutations (INDELs) and splice site mutations in the 27 PCNSL specimens. **(A)** INDELs detected in PCNSL specimens (N = 1). **(B)** Splice site mutations including SNV-types and INDEL-types detected in PCNSL specimens (N = 1). Mutation types were shown in the data matrix as missense, nonsense, stop-loss, INDELs including frameshift/non-frameshift with/without deletion or insertion, and splice site mutations, as the color configuration panel. Numbers on the right side of panels (N) indicate the numbers of specimens with SNVs and/or INDELs, and splice site mutations.

Cell growth-related genes

Symbol	Type	Sample 20	Sample 27	Sample 21	Sample 14	Sample 19	Sample 11	Sample 15	Sample 16	Sample 1	Sample 17	Sample 24	Sample 10	Sample 7	Sample 3	Sample 12	Sample 26	Sample 8	Sample 6	Sample 9	Sample 13	Sample 22	Sample 18	Sample 23	Sample 5	Sample 25	Sample 4	Sample 2	(N)	(Molecular functions)
CCND1	INDEL			7																									1	Cell cycle
CCND2	SNV		1																										1	Cell cycle
CDK6	SNV	1																											1	Cell cycle
MAP2K2	INDEL								4																				1	MAPK
MYCN	SNV									1																			1	Cell proliferation
NRAS	SNV						1																						1	Cell proliferation
SAMD9	INDEL											4																	1	Cell proliferation
TP53	SNV	1																											1	Apoptosis

Immune disease-related genes

Symbol	Type	Sample 20	Sample 27	Sample 21	Sample 14	Sample 19	Sample 11	Sample 15	Sample 16	Sample 1	Sample 17	Sample 24	Sample 10	Sample 7	Sample 3	Sample 12	Sample 26	Sample 8	Sample 6	Sample 9	Sample 13	Sample 22	Sample 18	Sample 23	Sample 5	Sample 25	Sample 4	Sample 2	(N)	(Molecular functions)
BCL6	SNV													1															1	NF-kB Signaling
FOXP1	SNV										1																		1	B-Cell Development
NFKB2	SNV		1																										1	NF-kB Signaling
PBX1	SNV	1																											1	Leukemia
TCF12	SNV		1																										1	B-Cell Development
TNFAIP3	SNV												1																1	NF-kB Signaling

Kinase genes

Symbol	Type	Sample 20	Sample 27	Sample 21	Sample 14	Sample 19	Sample 11	Sample 15	Sample 16	Sample 1	Sample 17	Sample 24	Sample 10	Sample 7	Sample 3	Sample 12	Sample 26	Sample 8	Sample 6	Sample 9	Sample 13	Sample 22	Sample 18	Sample 23	Sample 5	Sample 25	Sample 4	Sample 2	(N)	(Molecular functions)
ABL2	SNV						1																						1	non-RTK
ALK	SNV																1												1	non-RTK
ATR	SNV				1																								1	Ser/Thr Kinase
BRAF	SNV														3														1	Ser/Thr Kinase
EPHB6	SNV	1																											1	non-RTK
ERBB2	SNV							1																					1	RTK
FGFR2	SNV															1													1	RTK
IGF2R	SNV					1																							1	RTK
JAK1	SNV			1																									1	non-RTK
KIT	SNV					1																							1	RTK
RET	SNV			1																									1	RTK
ROS1	SNV				1																								1	RTK

1	missense
2	nonsense
3	stoploss
4	INDEL/frameshiftDeletion
5	INDEL/frameshiftInsertion
6	INDEL/nonframeshiftDeletion
7	INDEL/nonframeshiftInsertion

Supplementary Figure 7: Summary of significant SNVs and INDELs detected in genes related to cell growth, immune disease, and kinase in PCNSLs. Representative SNVs and INDELs are shown (N = 1). Cell growth-related genes, including cell proliferation, cell cycle, MAP-kinase, and apoptosis (upper). Immune disease-related genes, including NF-κB signaling, leukemia, and B-cell development and differentiation (middle). Kinase genes, including receptor tyrosine kinase (RTK), non-RTK, Ser/Thr kinase, and PI3-kinase (lower). Mutation types are shown in the data matrix as missense, nonsense, stop-loss, and INDEL mutations including frameshift/non-frameshift with/without deletion or insertion, as the color configuration panel. Numbers (N) at the right side of panels indicate the numbers of specimens detected.

A

gene	# locus	type	genotype	pvalue	allele_freq	function	protein	coding	Fwd_primer	Rev_Primer	sample
BCL10	chr1:85733383	SNV	T/G	1.00E-10	66.1	missense	p.Glu210Ala	c.629A>C	GTGTCATTGTCGTGAAACAGTACGTGA	TGAATTTGCCTGTTCTAGAAGTAGGCA	Sample 8
CD79B	chr17:62006799	SNV	A/T	1.00E-10	42.7	missense	p.Tyr197Asn	c.589T>A	GTCCTCATAGGTGGCTGTCTGGTCA	TGCACCTCACTCCTGACCCCTCAC	Sample 11
MYD88	chr3:38182641	SNV	T/C	2.00E-08	76.6	missense	p.Leu273Pro	c.818T>C	GAATGTGTGCCAGGGGTACTTAGAT	CTCAGAACAGTCTTCAGGGCA	Sample 14
MYH11	chr16:15847291	SNV	A/T	7.94E-09	56.7	missense	p.Asn615Lys	c.1845T>A	GGTGTTCAATTCTCCAGCTCAAAGCA	CTGGCCTTGGTTTCTAAGTTTCTAGT	Sample 20
PAX5	chr9:37002674	SNV	G/A	1.00E-10	41.6	missense	p.Ala192Val	c.575C>T	GGTGTTCGCGGGCACCTCTGCTGCCA	CTCCGCGCAGTGTCCACTGGCTCCGTGA	Sample 6
TET2	chr4:106196687	SNV	C/A	1.00E-09	47.1	missense	p.Pro1674Thr	c.5020C>A	AATACCCAATATCCATCATATCAATGCA	CATGATGTACATTTGGTCTAATGGTACA	Sample 24

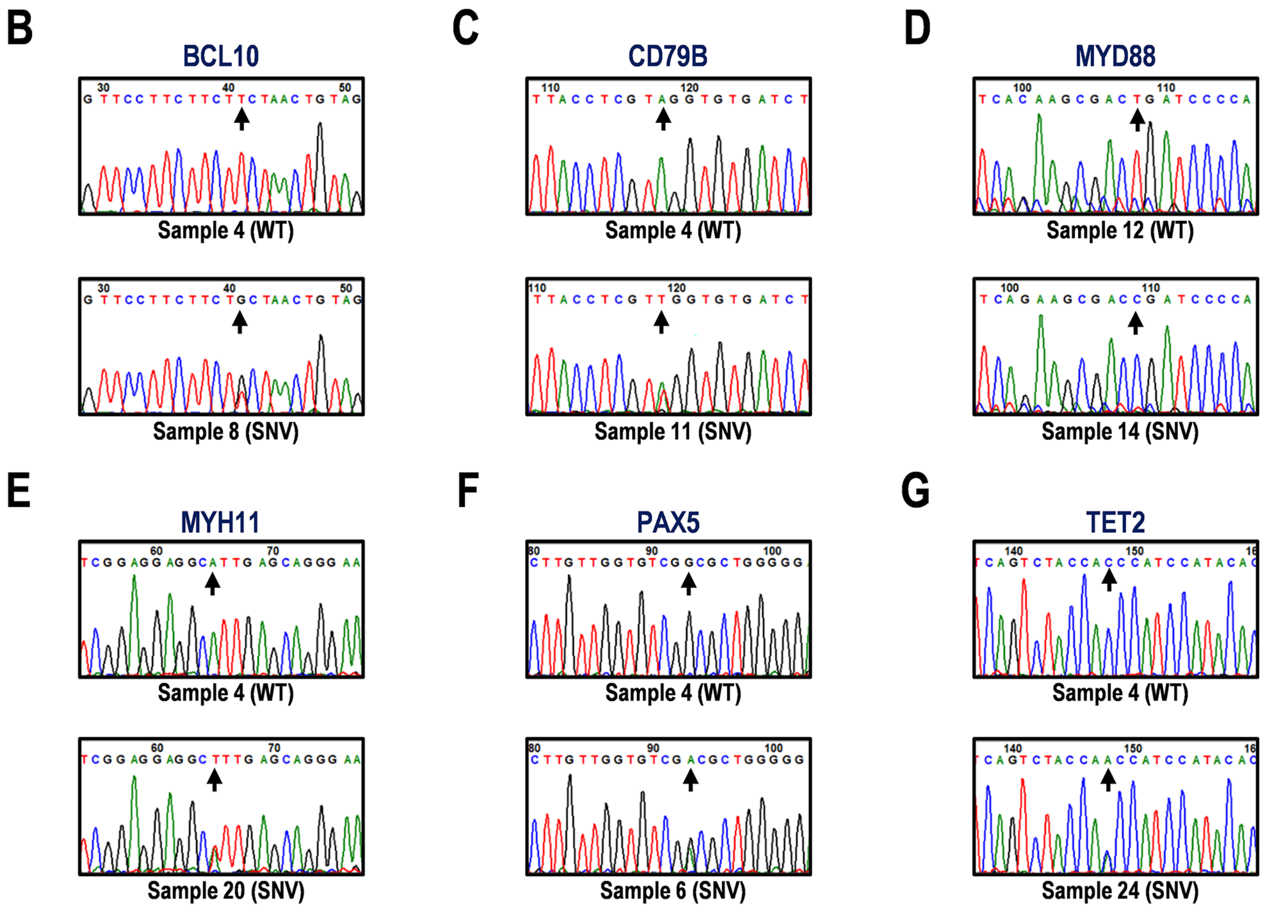

Supplementary Figure 8: Validation of the representative SNVs by Sanger sequencing. **(A)** Primers and representative SNVs in BCL10, CD79B, MYD88, MYH11, PAX5, and TET2 analyzed by Sanger sequencing. **(B-G)** Representative data for the sequencing results for the SNVs within BCL10 (B), CD79B (C), MYD88 (D), MYH11 (E), PAX5 (F), and TET2 (G).

A

		OS (month)		Univariate analysis		
		Median	95%CI	HR	95%CI	P-value
Total	27	53.43	25.13-73			
Male	13	59.46	16.6-NA	1		
Female	14	44.38	13.6-NA	0.92	0.35-2.37	0.86
Age < 50	4	115.5	28-115.5	1		
Age > 50	23	54.26	21.5-72.6	1.76	0.50-11.17	0.42

B

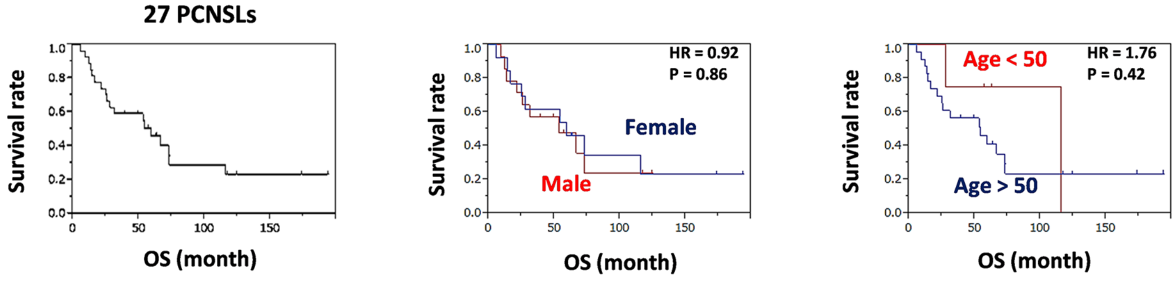

Supplementary Figure 9: Data set of the 27 PCNSL specimens in the study. **(A)** Statistics and univariate analysis for overall survival (OS) of 27 PCNSL patients examined. **(B)** Kaplan-Meier analysis for total specimens (left), and subgroups divided by gender (center) and age by 50 years (right) in 27 PCNSL patients examined. HR, hazard ratio.

A

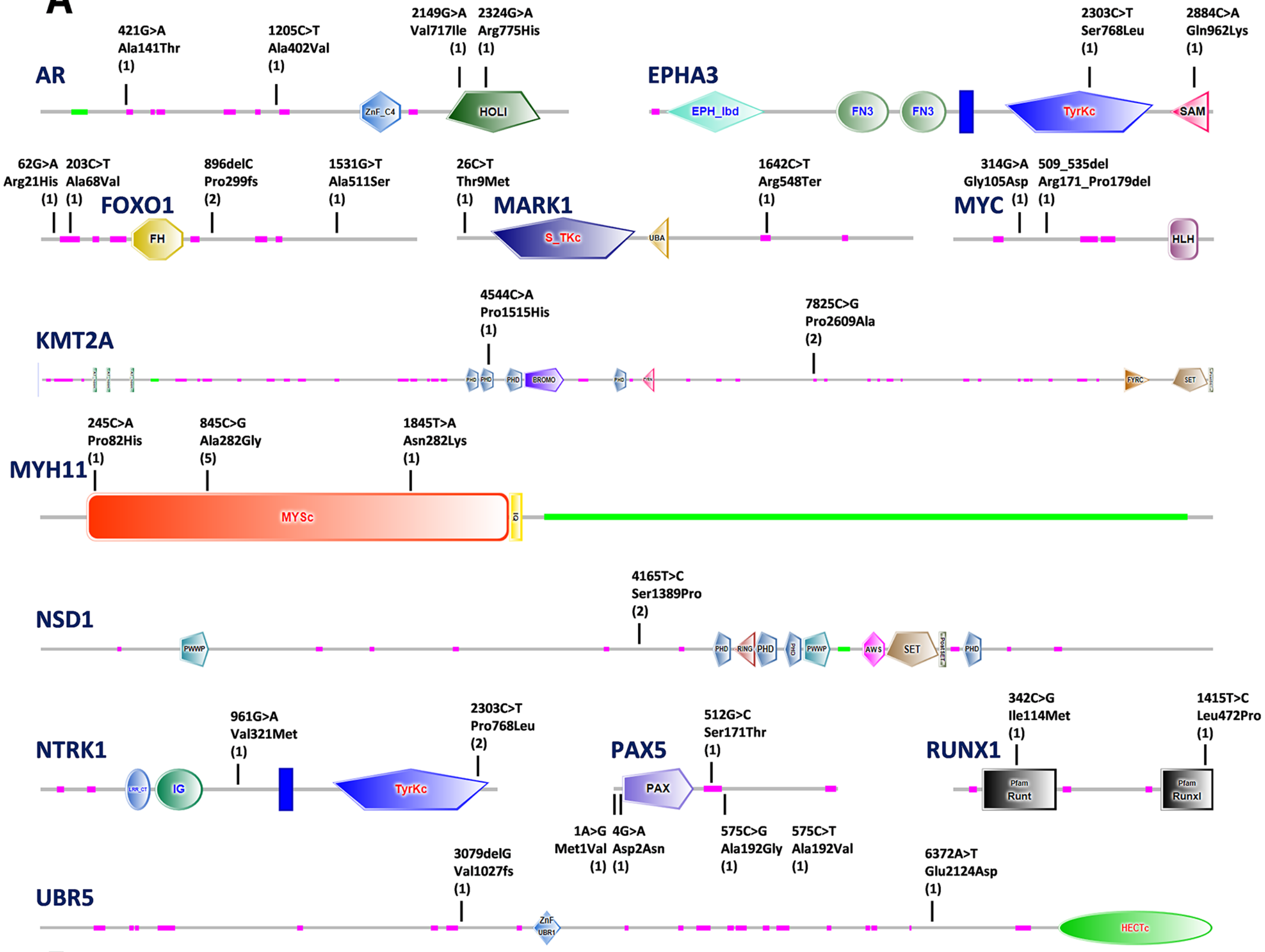

B

Gene	Refseq	Locus	Type	Coding	Protein	Pfam domain	P-value	Frequency	dbsnp
AR	NM_000044.3	ChrX:66931507	SNV	2149G>A	Val717Ile	HOLI	5.01E-08	42.7	
AR	NM_000044.3	ChrX:66941680	SNV	2324G>A	Arg775His	HOLI	1.26E-07	36.8	rs137852572
EPHA3	NM_005233.5	Chr3:89528584	SNV	2884C>A	Gln962Lys	SAM	2.00E-09	22.5	
EPHA3	NM_005233.5	Chr3:89480466	SNV	2303C>T	Ser768Leu	TyKc	7.94E-09	34.1	
KMT2A	NM_001197104.1	Chr11:118360571	SNV	4544C>A	Pro1515His	PHD	3.16E-09	18.1	
MYH11	NM_001040114.1	Chr16:15931865	SNV	245C>A	Pro82His	MYSc	1.58E-10	42.6	
MYH11	NM_001040114.1	Chr16:15847291	SNV	1845T>A	Asn615Lys	MYSc	7.94E-09	56.7	
MYH11	NM_001040114.1	Chr16:15870000	SNV	845C>G	Ala282Gly	MYSc	5.01E-08	16.0	
NTRK1	NM_002529.3	Chr1:156851346	SNV	2303C>T	Pro768Leu	TyKc	1.58E-07	61.7	
RUNX1	NM_001754.4	Chr21:36259149	SNV	342C>G	Ile114Met	Runt	1.00E-10	15.3	rs201747706
RUNX1	NM_001754.4	Chr21:36164460	SNV	1415T>C	Leu472Pro	Runxl	NA	17.8	

Supplementary Figure 10: Mutation distribution in the functional domains of the 12 candidates of diagnosis and prognosis marker genes in PCNSL. Candidate markers including AR, EPHA3, FOXO1, KMT2A, MARK1, MYC, MYH11, NSD1, NTRK1, PAX5, RUNX1, and UBR5. **(A)** Conserved domains were mapped using Simple Modular Architecture Research Tool (SMART; http://smart.embl-heidelberg.de/). Numbers in parentheses indicate the number of PCNSL specimens showing nucleotide alterations. **(B)** Summary of significant somatic mutations in functional domains predicted.

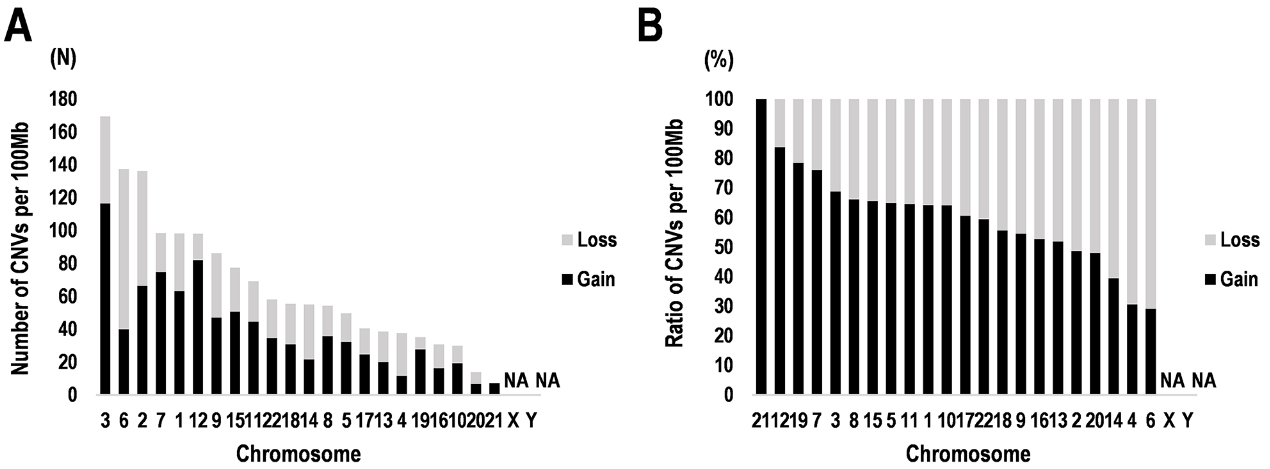

Supplementary Figure 11: Frequency of CNVs per 100 Mb in each chromosome in the 27 PCNSLs. (A) Numbers of CNVs per 100 Mb in each chromosome. **(B)** Ratios of gains and losses of CNVs per 100 Mb in each chromosome. NA, not applicable.

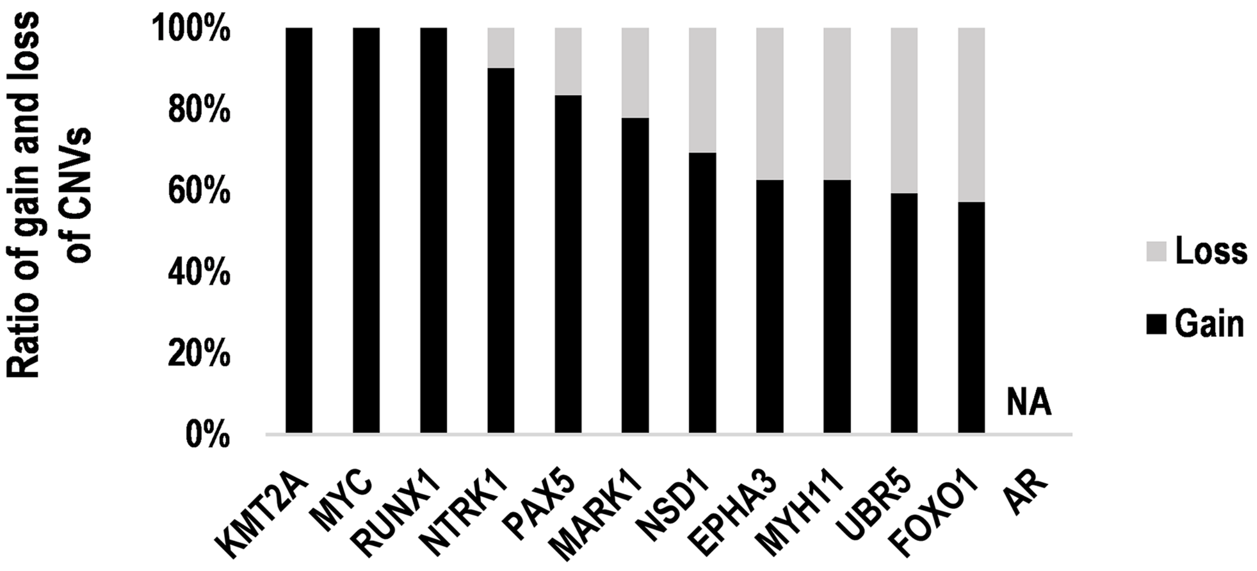

Supplementary Figure 12: Ratios of gains and losses of CNVs in the 27 PCNSL specimens. CNVs in AR were not detected. NA, not applicable.

A

		UBR5	FOXO1	MARK1	MYH11	NTRK1	EPHA3	KMT2A	PAX5	RUNX1	AR	MYC	NSD1
Group N≥1	Subgroup A	●	●	●	●	●	●	●	●	●	●	●	●
Group N≥1	Subgroup B												
Group N≥2	Subgroup A	●	●	●	●	●	●	●	●	●	●	●	●
Group N≥2	Subgroup B	●	●	●									
Group N≥3	Subgroup A	●	●	●	●	●	●	●	●	●	●	●	●
Group N≥3	Subgroup B	●	●	●	●	●							
Group N≥4	Subgroup A	●	●	●	●	●	●	●	●	●	●	●	●
Group N≥4	Subgroup B	●	●	●	●	●	●	●	●	●			

B

Group	Subgroup	N	Average OS (month)	95%CI	HR	95%CI	P-value
Group N≥1	A	21	54.09	33.62-74.55	1.243	0.439-4.435	0.6987
	B	6	74.03	8.25-139.81	1		
Group N≥2	A	18	54.5	30.51-78.49	1.527	0.574-4.773	0.4086
	B	9	66.55	26.71-106.39	1		
Group N≥3	A	16	54.64	27.29-82.00	2.007	0.754-6.274	0.1680
	B	11	64.16	32.79-95.52	1		
Group N≥4	A	9	54.04	10.10-97.97	1.838	0.699-4.680	0.2093
	B	18	60.76	38.03-83.49	1		

Supplementary Figure 13: (A) Combinations of the 12 candidate genes associated with SNVs and CNVs for PCNSL prognostic markers in the subgroups in each Kaplan-Meier survival analysis. Subgroups were divided according to two-way clustering analysis for the 12 candidates. Closed circles indicates "included." **(B)** Statistics for overall survivals (OS) of the PCNSL specimens in each analysis. Average of OS with 95%CI, hazard ratio (HR) with 95%CI, and log-rank test (P-value) in each analysis are presented.

A

Subgroup	N	OS (month) Median	95%CI	Multivariate analysis HR	95%CI	P-value
Gain(+)Loss(+)	8	28.18	12.26-66.7	3.488	1.169-11.183	0.0254
Gain(+)Loss(-)	14	72.6	28-NA	1		
Gain(-)Loss(+)	3	115.5	13.6-NA	1.173	0.171-5.126	0.847
Gain(+)	22	59.46	16.6-115.5	1.407	0.389-9.011	0.6394
Gain(-)	3	72.6	28-NA	1		
Loss(+)	11	31.23	14.23-72.6	2.445	0.897-7.276	0.0805
Loss(-)	14	115.5	13.6-NA	1		

B

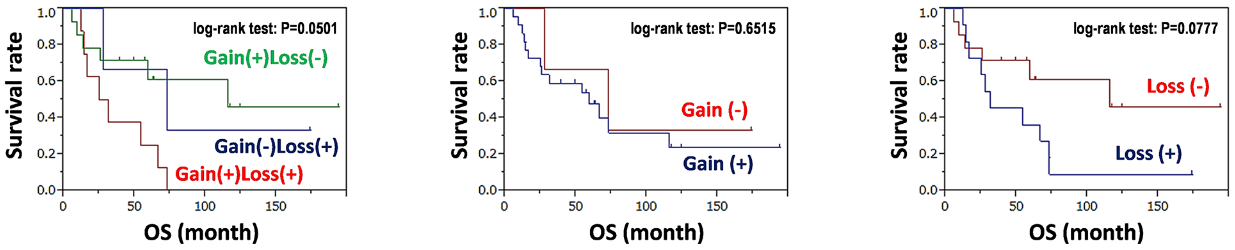

Supplementary Figure 14: Statistics and Kaplan-Meier survival analysis for upregulated oncopathways and downregulated proapoptotic pathways. **(A)** Statistics for OS and hazard ratios. **(B)** Kaplan-Meier analysis. The defined oncopathways include RTK-RAS-MAPK signaling, MYC, and the mTOR gene and the defined proapoptotic pathways include PTEN-PI3K-AKT signaling, the FAS death receptor gene, *FOXO1*, and the *p53* gene. The 25 PCNSL specimens were divided into two subgroups with or without the CNVs with gains of defined oncopathways and losses of the defined proapoptotic pathways. Two samples such as Samples 18 and 26 showing no CNVs in the genes of interest were excluded from Kaplan-Meier analyses.

Sample	Type	Location	Gain/Loss	Symbol
Sample 2	CNV	6q16.1(93953057-94120425)	1	EPHA7
Sample 24	CNV	7q21.2(91622270-91625126)	1	AKAP9
Sample 24	CNV	7q21.2(91630446-91632247)	5	AKAP9
Sample 24	CNV	7q21.2(91632247-91651635)	1	AKAP9
Sample 3	CNV	7q21.2(91641666-91652328)	5	AKAP9
Sample 4	CNV	7q21.2(91641666-91672127)	4	AKAP9
Sample 24	CNV	7q21.2(91651661-91670135)	4	AKAP9
Sample 3	CNV	7q21.2(91652328-91700334)	3	AKAP9
Sample 24	CNV	7q21.2(91670135-91672127)	1	AKAP9
Sample 4	CNV	7q21.2(91690689-92733085)	3	AKAP9|CDK6|SAMD9
Sample 3	CNV	7q21.2(91706946-91729215)	4	AKAP9
Sample 1	CNV	7q21.2(91706946-91739515)	3	AKAP9
Sample 12	CNV	7q21.2(91724312-92733085)	1	AKAP9|CDK6|SAMD9
Sample 3	CNV	7q21.2(91730141-92731040)	3	AKAP9|CYP51A1|LRRD1|KRIT1|ANKIB1|GATAD1|PEX1|RBM48|MGC16142|FAM133B|FAM133DP|CDK6|AC002454.1|SAMD9
Sample 17	CNV	7q21.2(91730141-92732987)	1	AKAP9|CYP51A1|LRRD1|KRIT1|ANKIB1|GATAD1|PEX1|RBM48|MGC16142|FAM133B|FAM133DP|CDK6|AC002454.1|SAMD9
Sample 14	CNV	7q31.2(116339126-116423527)	3	MET
Sample 2	CNV	7q31.2(116380037-116435976)	1	MET
Sample 1	CNV	9q21.2(80412352-80537268)	1	GNAQ
Sample 24	CNV	9q21.2q22.32(80336237-97873839)	3	GNAQ|SYK|FANCC
Sample 22	CNV	12q12(43747926-43763203)	8	ADAMTS20
Sample 22	CNV	12q12(43769121-46125175)	3	ADAMTS20|ARID2
Sample 12	CNV	12q12(43769803-43771388)	1	ADAMTS20
Sample 5	CNV	12q12(43777264-43828056)	3	ADAMTS20
Sample 12	CNV	12q12(43777264-43846413)	5	ADAMTS20
Sample 27	CNV	12q12(43777472-43840505)	4	ADAMTS20
Sample 6	CNV	12q12(43792793-43846197)	5	ADAMTS20
Sample 17	CNV	12q12(43792793-43846413)	4	ADAMTS20
Sample 24	CNV	12q12(43792793-43846413)	4	ADAMTS20
Sample 8	CNV	12q12(43828056-43828242)	10	ADAMTS20
Sample 5	CNV	12q12(43828056-43846413)	6	ADAMTS20
Sample 8	CNV	12q12(43833398-43846413)	4	ADAMTS20
Sample 27	CNV	12q12(43845982-46245168)	3	ADAMTS20|PUS7L|IRAK4|TWF1|TMEM117|NELL2|DBX2|RACGAP1P|PLEKHA8P1|RNY5|ANO6|LINC00938|ARID2
Sample 3	CNV	12q12(43846413-43856806)	1	ADAMTS20
Sample 7	CNV	12q12(43846413-43856806)	1	ADAMTS20
Sample 12	CNV	12q12(43846413-43858568)	1	ADAMTS20
Sample 3	CNV	12q12(43858381-46123712)	3	ADAMTS20|PUS7L|IRAK4|TWF1|TMEM117|NELL2|DBX2|RACGAP1P|PLEKHA8P1|RNY5|ANO6|LINC00938|ARID2
Sample 12	CNV	12q12(43944636-43944923)	1	ADAMTS20
Sample 12	CNV	12q12(43944923-46215305)	4	ADAMTS20|ARID2
Sample 6	CNV	12q12q14.1(43846197-58145560)	3	ADAMTS20|ARID2|KMT2D|ATF1|SMUG1|ERBB3|DDIT3|CDK4|MIR6759

Supplementary Figure 15: Summary of recurrent CNVs detected in 6q16.1, 7q21.2, 7q31.2, 9q21.2, and 12q12-14.1 in the 27 PCNSL specimens. CNV > 2: Gain (red), CNV < 2: Loss (green).

Supplementary Table 1: The results for PolyPhen-2 and SIFT analyses in the representative marker candidate genes.

		PolyPhen-2				SIFT			
	N	average	median	min	max	average	median	min	max
CD79B	5	1	1	1	1	0	0	0	0
EPHA3	1	1	1	1	1	0	0	0	0
KMT2A	1	1	1	1	1	0	0	0	0
NTRK1	1	1	1	1	1	0	0	0	0
TET2	1	1	1	1	1	0	0	0	0
AR	3	0.994667	0.995	0.989	1	0.003333	0	0	0.01
MAP2K2	1	0.994	0.994	0.994	0.994	0	0	0	0
EPHA7	1	0.98	0.98	0.98	0.98	0.05	0.05	0.05	0.05
MYD88	20	0.9695	1	0.39	1	0.0005	0	0	0.01
PDGFRB	2	0.947	0.947	0.894	1	0.03	0.03	0.03	0.03
MYCN	1	0.863	0.863	0.863	0.863	0.05	0.05	0.05	0.05
PIM1	27	0.853889	0.987	0.178	1	0.008519	0	0	0.05
MARK1	1	0.598	0.598	0.598	0.598	0	0	0	0
MYC	1	0.42	0.42	0.42	0.42	0	0	0	0
PAX5	2	0.285	0.285	0.247	0.323	0.015	0.015	0.01	0.02

N; the number of mutations with allele frequency > 0.15, NA; not applicable

Supplementary Table 2: Information for allele frequencies and SIFT and PolyPhen-2 analyses in the representative genes with low frequencies.

See Supplementary File 1
